# THF peroxide as a factor in generating desulphurised products from the solid-phase synthesis of phosphorothioate-modified oligonucleotides†

**DOI:** 10.1039/d4ra03592e

**Published:** 2024-07-08

**Authors:** Qin Ren, Takashi Osawa, Michiaki Tatsuno, Satoshi Obika

**Affiliations:** a Graduate School of Pharmaceutical Sciences, Osaka University 1-6 Yamadaoka Suita Osaka 565-0871 Japan; b Institute for Open and Transdisciplinary Research Initiatives, Osaka University 1-3 Yamadaoka Suita Osaka 565-0871 Japan

## Abstract

Antisense oligonucleotides (ASOs) are generally obtained *via* chemical synthesis on a solid support and phosphorothioate (PS) modification with a phosphate backbone to increase their *in vivo* stability and activity. However, desulphurised products, in which PS is partially replaced by phosphodiesters, are generally formed during the chemical synthesis of ASO and are difficult to separate from the desired PS-modified ASO by chromatography. Therefore, revealing the unknown factors that cause the formation of desulphurised products and proposing methods to inhibit their formation are highly desirable. In this study, it was found that peroxides in THF, which is used as a solvent for the acetyl capping agent, oxidise phosphite triesters to produce desulphurisation products. The use of THF with antioxidants effectively suppresses the oxidation caused by THF peroxides. Moreover, THF peroxide was found to oxidise phosphoramidites, which are the building blocks of oligonucleotide chemical syntheses, indicating that caution should be taken with the organic solvents used during the synthesis and purification of phosphoramidites.

## Abstract 2

The oxidation of phosphite triester by peroxides contained in THF as a solvent is reflected as a PO-substituted product during the synthesis of phosphorothioate-modified oligonucleotides.

## Graphical abstract

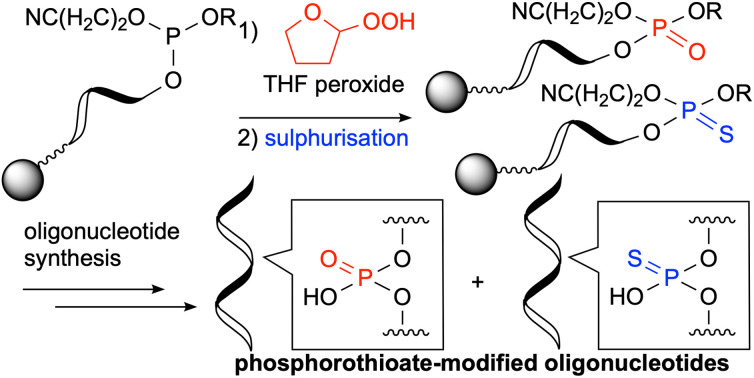

## Introduction

Oligonucleotide therapeutics have attracted considerable attention in recent years as a new drug discovery modality for genetic diseases that are difficult to target using conventional drug discovery methods. They are based on oligonucleotides of approximately 20 residues, and the majority of those that have been approved to date are antisense oligonucleotides (ASOs).^1^ For the practical application of ASOs, improving the *in vivo* stabilisation of oligonucleotides and their target RNA binding ability is crucial. To achieve this, various modified nucleic acids have been developed.^2–4^ For example, the phosphate backbone of ASO is chemically modified to protect it from *in vivo* nucleases that hydrolyse phosphodiester (PO) bonds. Typically, the *in vivo* stability of oligonucleotides can be significantly improved by phosphorothioate (PS) modification, in which the oxygen atom of the phosphate diester moiety is replaced with a sulphur atom.^5^ Furthermore, PS-modified oligonucleotides bind to plasma proteins, preventing renal excretion, and enhance interactions with cell surface proteins to promote the cellular uptake of ASOs.^6–8^ Because of these properties, PS modification has been used for most existing ASOs.

**Fig. 1:**
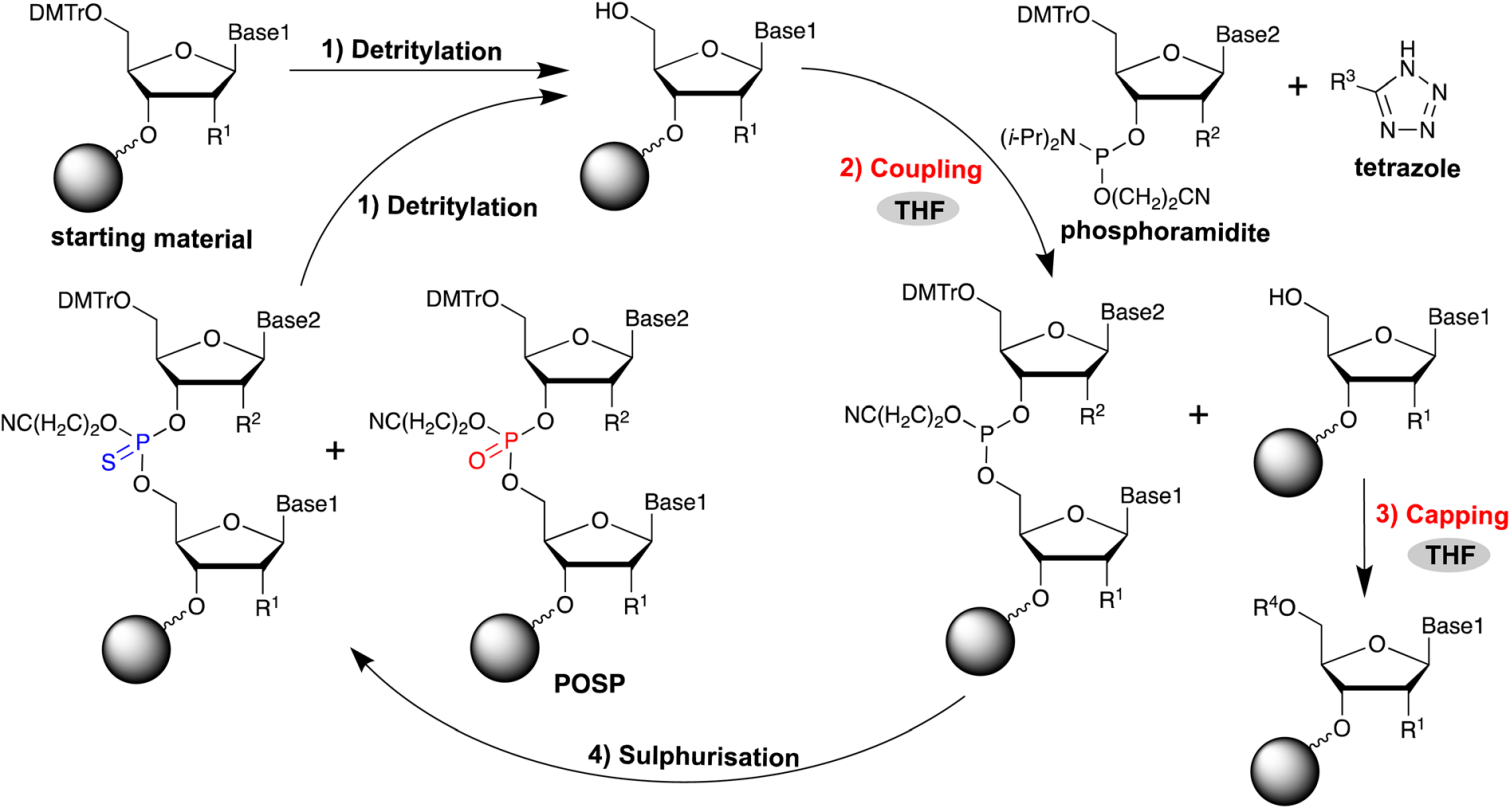
Solid-phase synthesis of oligonucleotides based on phosphoramidite chemistry and synthetic processes (coupling and capping), where the formation of PO-substituted products (POSPs) can occur owing to THF peroxide.

ASO is generally synthesised in the solid phase using the phosphoramidite method (Fig. 1).^9^ This method consists of four reactions: removal of the 5′-end DMTr group (detritylation); coupling of phosphoramidite to the 5′-OH group; acetylation of the unreacted 5′-OH group (acetyl capping); and oxidation of phosphite triester. The reaction cycle is repeated without purification until the oligonucleotides are elongated to the desired length. The introduction of PS modification in the phosphoramidite method can be achieved by oxidative sulphurisation of phosphite,^10–23^ which is the phosphoramidite coupling product. However, in the synthesis of PS-modified oligonucleotides, desulphurisation products in which PS is partially replaced by PO, referred to as PO-substituted products (POSPs), are often produced as major impurities in chemically synthesised PS-modified oligonucleotides.^24^ In addition, it is very difficult to maintain a high quality of PS-modified ASOs because the POSPs are very similar to the desired oligonucleotide in terms of molecular size and physical properties and are difficult to remove by High Performance Liquid Chromatography (HPLC) purification. Therefore, preventing its formation during oligonucleotide synthesis is necessary.

The desulphurisation of PS due to metal ions in aqueous ammonia,^25,26^ trace amounts of water in organic solvents,^27^ phosgene in aged trichloroacetic acid,^28^ ammonia treatment of alkylated thiophosphoric acid,^28^ and acetyl capping^29^ have been reported as factors in the formation of POSPs. However, despite the identification of these factors, completely suppressing the desulphurisation of PS using current ASO synthesis technology remains difficult. Therefore, we assumed that there may be reasons for the formation of POSPs other than the aforementioned factors. Very recently, we have developed a facile purification method for oligonucleotides that uses highly lipophilic phosphoramidites to protect unreacted 5′-OH groups and facilitate the removal of shortmer impurities resulting from nonquantitative coupling of phosphoramidites by simple reversed-phase chromatography.^30^ Cholesterol-phosphoramidite^31^ was used instead of acetylating agents, which was expected to suppress the formation of acetyl capping-derived POSPs. Based on this hypothesis, PS-modified oligonucleotides synthesised using our method were analysed and showed an unexpected increase in POSP. This suggests that the substitution of PS for PO occurred for a reason different from the known desulphurisation factors that have been identified. Comparing our method using cholesterol-phosphoramidite^30^ with the conventional conditions for oligonucleotide synthesis, cholesterol-phosphoramidite, THF, and tetrazoles were most likely involved in the formation of POSPs. In particular, THF is oxidised by oxygen in the air and converted to peroxides, depending on the storage conditions.^32^ Against this background, the desulphurisation of PS by THF peroxide, which proceeded with our simple purification method using cholesterol-phosphoramidite, was verified. In this study, we confirmed that THF peroxide increases the number of POSPs. Therefore, the relationship between the THF peroxides and the amount of POSP was investigated for the reagents used as THF solutions in the phosphoramidite method (Fig. 1). Furthermore, based on the findings, improvements to our simple purification method were made. This study presents the detailed results.

## Results and discussion

**Fig. 2:**
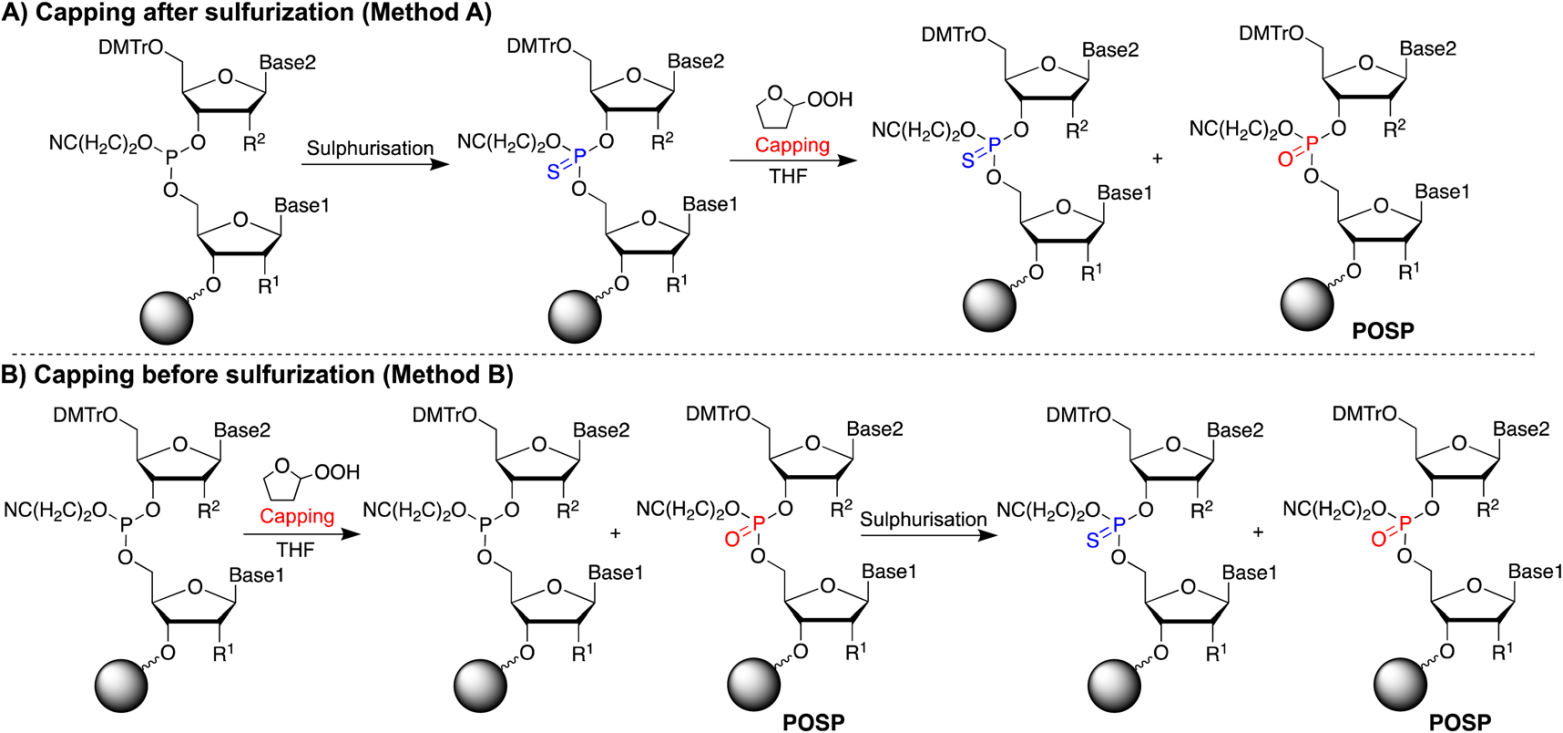
Hypothesis of generating POSPs by THF treatment. (A) Desulphurisation by capping after sulphurisation (Method A). (B) Oxidation of phosphite triester by capping before sulphurisation (Method B).

If THF peroxide generates POSPs, the following two pathways are reasonable (Fig. 2): first, PS is desulphurised using THF peroxide (Fig. 2A, Method A). Second, the phosphoramidite coupling product, the phosphite triester, may be oxidised by THF peroxide (Fig. 2B, Method B). To verify these two formation pathways, we first quantified the peroxides in several THFs with different post-opening storage times (Fig. S2†), referring to the paper by Beutner *et al.*^33^ The results showed that THF containing the stabiliser dibutylhydroxytoluene (BHT) contained negligible amounts of peroxide, less than 10 ppm (*ca.* 100 μM), and the longer after opening, the greater the amount of peroxide contained, which is consistent with the results in the literature.^33^

**Fig. 3:**
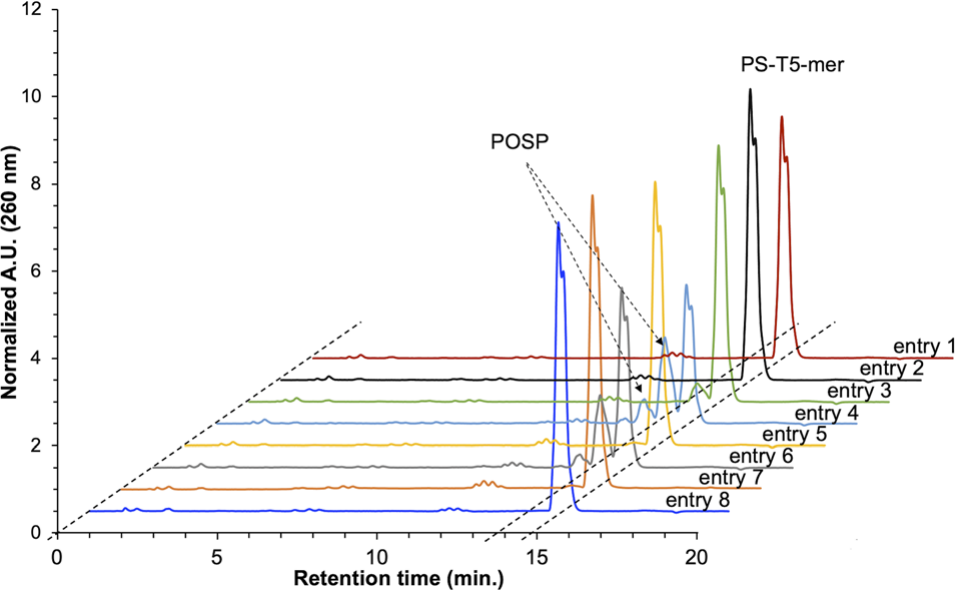
Reversed-phase HPLC charts of crude PS-T5-mer synthesised with or without the capping reagents shown in Table 1. HPLC column: waters XBridge® oligonucleotide BEH C18 column (4.6 × 50 mm), eluent A: H_2_O/HFIP/TEA = 100/1/0.1, eluent B: CH_3_OH, flow rate: 1.0 mL min^−1^, temperature: 50 °C, gradient: CH_3_OH 0–15% (20 min).

PS-modified T5-mer was subsequently synthesised using THF containing approximately 3135 ppm (*ca.* 30 mM) of peroxide and the capping conditions shown in Table 1. These samples were subjected to HPLC analysis, and the ratio of POSP formation was calculated from the peak areas of PS-T5mer and POSPs (Fig. 3). First, PS-T5-mer was synthesised without using a capping reagent, and no POSPs were observed (Table 1, entry 1). In addition, PS-T5-mer was synthesised using a THF solution of cholesterol-phosphoramidite and an acetonitrile solution of 5-ethylthio-1*H*-tetrazole (ETT) as capping reagents before sulphurisation. Consequently, similar to that without capping reagents, no POSPs were generated (Table 1, entry 2). This means that it is very unlikely that PS is desulphurised by THF peroxide, one of the two expected formation pathways of POSPs shown in Fig. 2A. Next, 7.7% of the POSPs were generated when cholesterol-phosphoramidite and ETT were pumped into the solid phase prior to the sulphurisation of the phosphite triester (Table 1, entry 3). In our recently reported purification method,^30^ the capping of the 5′-OH group by cholesterol-phosphoramidite was performed before sulphurisation of the phosphite triester. Therefore, the result shown in entry 3 is consistent with our recent results. As noted above, PS is probably not desulphurised by THF peroxide; thus, the results of entry 3 suggest that phosphite triester may have been oxidised by THF peroxide, as shown in Fig. 2B.

**Table 1:** Relationship between the capping conditions and the POSP ratio in PS-T5-mer synthesis

Entry	Capping reagents	THF peroxide (mM)	Method^a^	POSPs^b^ (%)
1	None	—	—	0
2	Cholesterol-phosphoramidite in THF, ETT in MeCN	30	A	0
3	Cholesterol-phosphoramidite in THF, ETT in MeCN	30	B	7.7
4	THF, ETT in MeCN	30	B	47.2
5	MeCN, ETT in MeCN	—	B	1.1
6	THF	30	B	34.6
7	THF with BHT	<0.1	B	0.9
8	Ac_2_O, pyridine in THF with BHT, 1-methylimidazole in THF with BHT	<0.1	B	0.5

a Method A and B are shown in Fig. 2.

b Conversion rate to POSP calculated from UV area ratio of HPLC analysis.

From these results, conditions without cholesterol-phosphoramidite or ETT were investigated to show a dramatic increase in the ratio of POSPs (Table 1, entries 4 and 6). In contrast, when THF containing BHT with a THF peroxide concentration of less than 10 ppm (*ca.* 100 μM) was used, few POSPs were produced (Table 1, entry 7). Furthermore, substituting THF for MeCN resulted in negligible phosphite triester oxidation (Table 1, entry 5). Based on these results, PS-T5-mer was synthesised using THF with adjusted concentrations of THF peroxide instead of a capping reagent. The results showed a concentration-dependent increase in the percentage of POSPs (Table S2 and Fig. S3†), clearly indicating that THF peroxide is a factor in the formation of POSPs. In contrast, since phosphoramidite is a trivalent phosphorus compound like phosphite triester, the fact that only 7.7% of POSPs were generated under the entry 3 condition may be attributed to the consumption of THF peroxide by the oxidation of cholesterol-phosphoramidite. In addition, few POSPs were observed when PS-T5-mer was synthesised by the conventional acetyl capping method (Table 1, entry 8). The THFs used in commercially available acetic anhydride and 1-methylimidazole solutions contain BHT, which may be the reason why almost no PO-substitution of PS occurred.

The contribution of THF peroxides to the formation of POSPs during conventional oligonucleotide synthesis was examined. For example, phosphoramidites are generally used in acetonitrile solutions, whereas some chemically modified artificial nucleic acid phosphoramidites are less soluble in acetonitrile and are used as THF solutions.^34,35^ In addition, acetyl capping reagents are often used as THF solutions. As mentioned earlier, because the THF solvent of the commercial acetyl capping reagent contains antioxidants such as BHT, very few POSPs were produced (Table 1, entry 8). However, because the complete suppression of the formation of THF peroxides is difficult even in the presence of antioxidants, the possibility that old capping reagents with insufficient control may contain THF peroxides cannot be ruled out. Therefore, the relationship between THF peroxide and the amount of POSPs in the phosphoramidite coupling and acetyl capping processes was investigated (Table 2). First, PS-T5-mer was synthesised using thymidine-phosphoramidite dissolved in THF containing 483 ppm (*ca.* 4.6 mM) of peroxide or in a mixture of THF and acetonitrile (1 : 3). The results showed no significant increase in the formation of POSPs when the phosphoramidite solvent was changed to THF (Table 2, entries 1–5, and Fig. S4†). This suggests that the peroxide in the THF solution was almost completely consumed by the oxidation of thymidine-phosphoramidite and cholesterol-phosphoramidite. Therefore, thymidine phosphoramidite was dissolved in THF containing 1336 ppm (*ca.* 13 mM) of peroxide to a final concentration of 67 mM, and the changes over time were analysed by LC/MS. The reaction was completed in 5 min, and a peak corresponding to approximately 10% of the oxidised product with a molecular weight increase of 16 was observed (Fig. S5†), indicating that THF peroxide can oxidise phosphoramidite.

**Table 2:** Ratio of POSP formation in PS-T5-mer synthesis using thymidine phosphoramidite dissolved in THF containing peroxide

Entry	Solvents	BHT	THF peroxide (mM)	POSPs^a^ (%)
1	MeCN	—	0	2.5
2	25% THF in MeCN	—	<0.1	2.4
3	25% THF in MeCN	○	4.6	2.3
4	THF	—	<0.1	3.3
5	THF	○	4.6	2.5

a Conversion ratio to POSP was calculated from the UV area ratio of the HPLC analysis.

In contrast, the synthetic conditions using THF as a solvent for phosphoramidite resulted in the increased formation of shortmers, such as PS-T4-mer, compared to the general synthetic conditions using MeCN (Fig. S4 and Table S3†). Because the reaction rate of phosphoramidite and tetrazoles in THF, which is the rate-limiting step of the coupling reaction, is slower than that in MeCN,^36^ the use of THF as a solvent reduces the coupling efficiency of phosphoramidite, which may be the reason for the increased amount of shortmers produced. Although the phosphoramidite concentration decreased by the oxidation of phosphoramidite, almost no difference in the amount of shortmers produced was observed with and without BHT (Fig. S4 and Table S3†). Based on these results, when THF is used as a phosphoramidite solvent, the decrease in the coupling efficiency of phosphoramidite during the formation of POSPs should be focused on. In contrast, THF is sometimes used as a reaction solvent for the phosphitylation of chemically modified nucleic acids.^37–41^ The above experimental results indicate that phosphoramidites may be oxidised by THF peroxide even during their synthesis, leading to yield loss. In addition, common ethers, such as diethyl ether, which are used for the extraction and recrystallisation of organic compounds, are also known to contain peroxides,^42^ and care should be taken with the solvents used in the purification of phosphoramidite after the reaction.

As a model for older acetyl-capping reagents that may contain THF peroxides, PS-T5-mer and nusinersen (Fig. S6†) were synthesised using acetyl-capping reagents prepared with THF containing 507 ppm (*ca.* 4.9 mM) of peroxide and commercially available reagents containing BHT (Table 3, Fig. S7, and S8†). The results showed that capping before sulphurisation (Fig. 2B, Method B) tended to increase the amount of POSPs compared to capping after sulphurisation (Fig. 2A, Method A) in THF solutions of acetic anhydride or 1-methylimidazole with little peroxide (Table 3, entries 1 and 2). In addition, nusinersen, presumably because of its longer base than the T5-mer, was exposed to the acetylation reagent more frequently, resulting in a few percent increase in POSPs, which was more pronounced when the acetylation reagent was from a THF solution containing 507 ppm (*ca.* 4.9 mM) of peroxide. Specifically, when capping was performed before sulphurisation, the percentages of POSPs were 2.4% for the T5-mer and 16.4% for nusinersen (Table 3, entry 3). In contrast, when capping was performed after sulphurisation, the percentage of POSPs was only 2%, even for nusinersen, and very few POSPs were produced in the case of the T5-mer (Table 3, entry 4). These results indicate that peroxides in THF, which is the solvent of the acetylation reagent, are factors in the formation of POSPs and that acetylation should be performed after sulphurisation to inhibit the formation of POSPs. However, it is probably best not to use THF as the solvent for the acetylation reagent because it could be difficult to completely inhibit POSP formation caused by THF peroxide. In other words, the use of a peroxide-free solvent, such as MeCN, may be effective in inhibiting POSP formation. For example, MeCN solutions of 1-methylimidazole and acetic anhydride are commercially available, and their use is expected to improve the control of POSP formation. Entries 4 and 5 in Table 1 show that, using MeCN as an alternative solvent to THF, the ratio of POSPs decreased significantly, which supports this prediction.

**Table 3:** Effect of the acetyl capping agent containing THF peroxide on POSP formation

Entry	Capping reagents	BHT	THF peroxide (mM)	POSPs (%)	
				PS-T5-mer	Nusinersen
1	Ac_2_O, pyridine in THF, 1-methylimidazole in THF (Method B in Fig. 2)	○	<0.1	0.5^a^	3.0^b^
2	Ac_2_O, pyridine in THF, 1-methylimidazole in THF (Method A in Fig. 2)	○	<0.1	0^a^	1.6^b^
3	Ac_2_O, pyridine in THF, 1-methylimidazole in THF (Method B in Fig. 2)	—	4.9	2.4^a^	16.4^b^
4	Ac_2_O, pyridine in THF, 1-methylimidazole in THF (Method A in Fig. 2)	—	4.9	0^a^	1.9^b^

a Conversion ratio to POSP was calculated from the UV area ratio of the HPLC analysis.

b Conversion rate to POSP calculated from the MS intensity ratio of LC/MS analysis.

Finally, we verified whether the use of THF containing an antioxidant in our purification method using cholesterol-phosphoramidite could suppress the formation of POSPs (Table 4, Fig. S9, and S10†). The ratio of POSPs was 2.4% for the T5-mer and 17.8% for nusinersen in the synthesis using cholesterol-phosphoramidite dissolved in THF containing 507 ppm (*ca.* 4.9 mM) of peroxide (Table 4, entry 1). When cholesterol-phosphoramidite was dissolved in THF containing BHT, the formation of POSPs was reduced by less than a quarter compared to that in THF without BHT (Table 4, entry 2). A purification method that we have reported very recently allows for the removal of all shortmers due to nonquantitative coupling of phosphoramidites from the desired oligonucleotides by simple and easy reversed-phase chromatography using a short column for HPLC pretreatment.^30^ The results shown in Table 4 indicate that the use of THF containing the antioxidant BHT and the cholesterol modification prior to sulphurisation (Fig. 2B, Method B) can inhibit POSP formation and conveniently remove shortmer impurities from the desired oligonucleotides. However, the fact that POSP formation could not be completely suppressed in this experiment, as in the aforementioned experiment with the acetylating agent (Table 3), is probably due to the fact that the THF containing the antioxidant also contains a very small amount of peroxide. Using the same approach for solvent selection in the acetylating agent, it would be best not to use THF in our simple purification of oligonucleotides synthesised using cholesterol-phosphoramidite. However, cholesterol-phosphoramidite can be dissolved in THF because of its low solubility in MeCN.^30^ On the other hand, in the same paper on our simplified purification, stearyl alcohol modification could increase the retention of oligonucleotides in reversed-phase columns almost as much as cholesterol modification. Because the phosphoramidite of stearyl alcohol is soluble in MeCN/CH_2_Cl_2_ (1 : 3),^43^ the use of phosphoramidites of linear alkyl alcohols consisting of 20–30 carbon atoms would further suppress the formation of POSPs. Additionally, LC-MS data revealed that *ca.* 5% of the cyanoethyl adducts were generated during the synthesis of nusinersen using the acetyl capping method (Fig. S8†), whereas almost no cyanoethyl adducts were observed in the crude nusinersen synthesised using the cholesterol-capping method (Fig. S10†). Although a cyanoethyl group is added to the N3-position of thymine under the strongly basic conditions of treatment with aqueous ammonia,^44^ the cyanoethyl adduct produced in this experiment may have been derived from acetyl capping. Therefore, future experiments should clarify the factors that lead to the formation of cyanoethyl adducts.

**Table 4:** Effect of cholesterol-phosphoramidite solution with THF peroxide on POSP formation

Entry	Capping reagents	BHT	THF peroxide	POSPs (%)	
			(mM)	PS-T5-mer	Nusinersen
1	Cholesterol-phosphoramidite in THF ETT in MeCN	—	4.9	4.1^a^	17.8^b^
2	Cholesterol-phosphoramidite in THF ETT in MeCN	○	<0.1	0.7^a^	4.0^b^

a Conversion rate to POSP calculated from the UV area ratio in HPLC analysis.

b Conversion rate to POSP calculated from the MS intensity ratio of LC/MS analysis.

## Conclusions

In this study, PS-T5mer and nusinersen were synthesised, and the correlation between the POSP and the amount of peroxide in THF used in their synthesis was investigated in detail. The results showed that the peroxide in THF oxidises the phosphite triester, which is the coupling product of phosphoramidite chemistry. In addition, phosphoramidite can be oxidised by THF peroxide. Based on these results, reaction and purification solvents that are as peroxide-free as possible should be selected for phosphoramidite synthesis, and particular care should be taken when using THF. In oligonucleotide synthesis, peroxides in THF, which is the solvent for acetyl capping reagents and cholesterol-phosphoramidite, were found to be possible factors in the formation of POSPs. However, they could not be completely suppressed using THF containing BHT, which is an antioxidant. To completely suppress the formation of POSP, an alternative solvent, such as MeCN, should be used instead of THF. Our results are not only beneficial for suppressing impurities in chemically synthesised PS-modified ASOs but also provide guidelines for the selection of organic solvents to improve phosphoramidite quality and reaction yields.

## Experimental

### General

Phosphoramidites of 2′-methoxyethyl-RNA were purchased from Glen Research, and dT-phosphoramidite, Bz-dC-phosphoramidite, and iBu-dG-phosphoramidite were purchased from Sigma-Aldrich. Cholesterol-phosphoramidites were prepared according to the literature.^31^ THF, with or without BHT, was purchased from Fujifilm Wako Chemicals. Other chemical reagents and solvents were purchased from Fujifilm Wako Chemicals and Tokyo Chemical Industry (TCI). The oligonucleotides were synthesised using an automated DNA synthesiser (Nihon Techno Service, T-2-TRS). For HPLC, CBM-20A, DGU-20A, LC-20AD, CTO-20AC, SPD-20A, FRC-10A, and SIL-20A columns (SHIMADZU) were used. For the analysis column, the Waters XBridge® oligonucleotide BEH C18 (2.5 μm, 4.6 × 50 mm) was used. For LC-MS analysis, a Waters XeVO G2-XS QTof was used, and the obtained data were analysed using ProMass for MassLynx.

### Quantitation of THF peroxide

The quantitation of THF peroxide was performed according to the protocol presented in the literature.^33^ Benzyl phenyl sulphide (100 mg) was dissolved in MeCN (1.0 mL), and then vanadyl isopropoxide (VO(OiPr)_3_, 5.0 μL) was added. Fifty microliters of this solution were mixed with THF (950 μL), and the mixture was allowed to react at room temperature for 3 h without stirring. A portion of the reaction solution (10 μL) was withdrawn and analysed using HPLC. THF peroxide was quantified from the obtained UV peak area of benzyl phenyl sulfoxide, and a calibration curve was prepared in advance using *tert*-butyl hydroperoxide (TBHP, Fig. S1†).

### Oligonucleotide synthesis

All the phosphoramidites, except cholesterol-phosphoramidite, were dissolved in anhydrous MeCN to a final concentration of 0.067 M. Cholesterol-phosphoramidite was dissolved in anhydrous THF or anhydrous THF with BHT to a final concentration of 0.067 M. As shown in Table 2, a thymidine-phosphoramidite was dissolved in THF or THF/MeCN (1/3). The synthesis of oligonucleotides was performed on a 0.2 μmol scale by using an automated DNA synthesiser with 0.25 M 5-(ethylthio)-1*H*-tetrazole in MeCN as an activator, 3% trichloroacetic acid in CH_2_Cl_2_ as a deblocking reagent, and 0.05 M DDTT in pyridine/MeCN as a sulphurising reagent. Commercially available THF/pyridine/Ac_2_O and 16% 1-methylimidazole in THF were used as acetyl-capping reagents, as shown in Tables 1–3 (entries 1 and 2). For the synthesis of the oligonucleotides shown in entries 3 and 4 of Table 3, THF/pyridine/Ac_2_O and 16% 1-methylimidazole in THF were prepared by mixing commercial chemical reagents. The oligonucleotides synthesised in trityl-off mode were cleaved from the CPG resin, and all protecting groups were removed by treatment with 28% NH_4_OH.

### HPLC analysis

Oligonucleotides were analysed using a reversed-phase column with a mixture of H_2_O/HFIP/Et_3_N (100/1/0.1) as eluent A and MeOH as eluent B. A linear gradient from 0% to 15% MeOH (over 20 min) was applied at a flow rate of 1 mL min^−1^ and a temperature of 50 °C. The process was monitored by UV visualisation at a wavelength of 260 nm.

### LC/MS analysis

Crude oligonucleotides (PS-T5-mer and nusinersen) synthesised by acetyl or cholesterol-phosphoramidite capping were analysed using a reversed-phase column with a mixture of H_2_O/HFIP/Et_3_N (100/1/0.1) as eluent A and MeOH as eluent B. A linear gradient from 5% to 70% MeOH (over 10 min) was used at 50 °C at a flow rate of 0.3 mL min^−1^. The process was monitored by UV visualisation at a wavelength of 260 nm.

## Data availability

The authors confirm that the data supporting the findings of this study are available within the article or its ESI.†

## Author contributions

Q. Ren and M. Tatsuno conducted oligonucleotide synthesis and analysis. T. Osawa conceived the study. S. Obika supervised the study and provided financial support. Q. Ren and T. Osawa wrote and edited the manuscript. All the authors read the manuscript and agreed to its contents.

## Conflicts of interest

There are no conflicts to declare.

## Supplementary Material

RA-014-D4RA03592E-s001

## References

[cit1] Moumné L., Marie A. C., Crouvezier N. (2022). Pharmaceutics.

[cit2] McKenzie L. K., El-Khoury R., Thorpe J. D., Damha M. J., Hollenstein M. (2021). Chem. Soc. Rev..

[cit3] Khvorova A., Watts J. K. (2017). Nat. Biotechnol..

[cit4] Wan W., Seth P. P. (2016). J. Med. Chem..

[cit5] Eckstein F. (2014). Nucleic Acid Ther..

[cit6] Bijsterbosch M. K., Manoharan M., Rump E. T., De Vrueh R. L. A., van Veghel R., Tivel K. L., Biessen E. A. L., Bennett C. F., Cook P. D., van Berkel T. J. C. (1997). Nucleic Acids Res..

[cit7] Ezzat K., Aoki Y., Koo T., McClorey G., Benner L., Corenen-Stass A., O'Donovan L., Lehto T., Garcia-Guerra A., Nordin J., Saleh A. F., Behlke M., Morris J., Goyenvalle A., Dugovic B., Leumann C., Gordon S., Gait M. J., El-Andaloussi S., Wood M. J. A. (2015). Nano Lett..

[cit8] Miller C. M., Donner A. J., Blank E. E., Egger A. W., Kellar B. M., Østergaard M. E., Seth P. P., Harris E. N. (2016). Nucleic Acids Res..

[cit9] Beaucage S. L., Iyer R. P. (1992). Tetrahedron.

[cit10] Kamer P. C. J., Roelen H. C. P. F., van den Elst H., van der Marel G. A., van Boom J. H. (1989). Tetrahedron Lett..

[cit11] Iyer R. P., Phillips L. R., Egan W., Regan J. B., Beaucage S. L. (1990). J. Org. Chem..

[cit12] Vu H., Hirschbein B. L. (1991). Tetrahedron Lett..

[cit13] Rao M. V., Reese C. B., Zhao Z. (1992). Tetrahedron Lett..

[cit14] Stec W. J., Uznanski B., Wilk A. (1993). Tetrahedron Lett..

[cit15] Rao M. V., Macfarlane K. (1994). Tetrahedron Lett..

[cit16] Efimov V. A., Kalinkina A. L., Chakhmakhcheva O. G., Hill T. S., Jayaraman K. (1995). Nucleic Acids Res..

[cit17] Xu Q., Musier-Forsyth K., Hammer R. P., Barany G. (1996). Nucleic Acids Res..

[cit18] Zhang Z., Nichols A., Alsbeti M., Tang J. X., Tang J. Y. (1998). Tetrahedron Lett..

[cit19] Zhang Z., Nichols A., Tang J. X., Han Y., Tang J. Y. (1999). Tetrahedron Lett..

[cit20] Tang J. Y., Han Z., Tang J. X., Zhang Z. (2000). Org. Process Res. Dev..

[cit21] Krotz A. H., Gorman D., Mataruse P., Foster C., Godbout J. D., Coffin C. C., Scozzari A. N. (2004). Org. Process Res. Dev..

[cit22] Hanusek J., Russell M. A., Laws A. P., Jansa P., Atherton J. H., Fettesd K., Page M. I. (2007). Org. Biomol. Chem..

[cit23] Scotson J. L., Andrews B. I., Laws A. P., Page M. I. (2016). Org. Biomol. Chem..

[cit24] Bergot B. J., Egan W. (1992). J. Chromatogr..

[cit25] Kodra J. T., Kehler J., Dahl O. (1995). Nucleic Acid Res..

[cit26] Reese C. B., Song Q. (1997). Nucleic Acid Res..

[cit27] Ghisaidoobe A. B. T., de Koning M. C., Duynstee H. I., Ten Kortenaar P. B. W., Overkleeft H. S., Filippov D. V., van der Marel G. A. (2008). Tetrahedron Lett..

[cit28] Cieślak J., Ausín C., Chmielewski M. K., Kauffman J. S., Snyder J., Del-Grosso A., Beaucage S. L. (2005). J. Org. Chem..

[cit29] Cedillo I. E. , Janczak D. and Weaver P. M., US Pat., US2015021820A1, 2015

[cit30] Ren Q., Osawa T., Obika S. (2024). Tetrahedron.

[cit31] Seo Y. J., Jeong H. S., Bang E.-K., Hwang G. T., Jung J. H., Jang S. K., Kim B. H. (2006). Bioconjugate Chem..

[cit32] Ooizumi N., Yoshino S., Kumasaki M., Miyake A. (2011). Sci. Technol. Energ. Mater..

[cit33] Beutner G. L., Ayers S., Chen T., Leung S. W., Tai H. C., Wang Q. (2020). Org. Process Res. Dev..

[cit34] Osawa T., Hitomi Y., Wakita S., Kim H., Ito Y., Hari Y. (2018). Bioorg. Med. Chem..

[cit35] Koshkin A. A., Singh S. K., Nielsen P., Rajwanshi V. K., Kumar R., Meldgaard M., Olsen C. E., Wengel J. (1998). Tetrahedron.

[cit36] Nurminen E. J., Mattinenm J. K., Lonnberg H. J. (1999). J. Chem. Soc., Perkin Trans. 2.

[cit37] Yahara A., Shrestha A. R., Yamamoto T., Hari Y., Osawa T., Yamaguchi M., Obika S. (2012). ChemBioChem.

[cit38] Hari Y., Osawa T., Kotobuki Y., Yahara A., Shrestha A. R., Obika S. (2013). Bioorg. Med. Chem..

[cit39] Karale K., Bollmark M., Stulz R., Honcharenko D., Tedebark U., Strömberg R. (2021). Molecules.

[cit40] Maydanovych O., Easterwood L. M., Cui T., Véliz E. A., Pokharel S., Beal P. A. (2007). Methods Enzymol..

[cit41] Palframan M. J., Alharthy R. D., Powalowska P. K., Hayes C. J. (2016). Org. Biomol. Chem..

[cit42] Tommaso S. D., Rotureau P., Crescenzi O., Adamo C. (2011). Phys. Chem. Chem. Phys..

[cit43] Glen Report 24.17: New Product – 5'-Stearyl Phosphoramidite

[cit44] Capaldi D. C., Gaus H., Krotz A. H., Arnold J., Carty R. L., Moore M. N., Scozzari A. N., Lowery K., Cole D. L., Ravikumar V. T. (2003). Org. Process Res. Dev..

