# Supplementary material for: THF peroxide as a factor in generating desulphurised products from the solid-phase synthesis of phosphorothioate-modified oligonucleotides
Source: RSC Adv. 2024 Jul 8;14(30):21590–6. doi: 10.1039/d4ra03592e (PMC11229082; doi:10.1039/d4ra03592e)
Supplement: RA-014-D4RA03592E-s001 [file RA-014-D4RA03592E-s001.pdf]

Electronic Supplementary Material (ESI) for RSC Advances.
This journal is © The Royal Society of Chemistry 2024

Supplementary Information

THF peroxide as a factor in generating desulphurised products from the solid-phase synthesis of phosphorothioate-modified oligonucleotides

Qin Ren,†[a] Takashi Osawa,†[a] Michiaki Tatsuno [a] and Satoshi Obika *[a,b]

[a] Graduate School of Pharmaceutical Sciences, Osaka University, 1-6 Yamadaoka, Suita, Osaka 565-0871, Japan

[b] Institute for Open and Transdisciplinary Research Initiatives, Osaka University, 1-3 Yamadaoka, Suita, Osaka 565-0871, Japan

E-mail: obika@phs.osaka-u.ac.jp (S.Obika).

Contents

1. Determination of peroxide concentration in THF used in this study S2–S3

2. Relationship between capping conditions and POSP ratio in PS-T5-mer synthesis S4

3. Impurities contained in PS-T5-mer synthesised using dT phosphoramidite in THF S5

4. Oxidation of thimidine-phophoramidite by THF peroxide S6

5. Structure of nusinersen S7

6. POSP ratios contained in oligonucleotides synthesised by acetyl capping method S8

7. POSP ratios contained in oligonucleotides synthesised by cholesterol capping method S9

8. LC/MS data of oligonucleotides S10– S25

1. Determination of peroxide concentration in THF used in this study

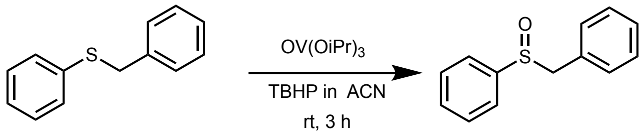

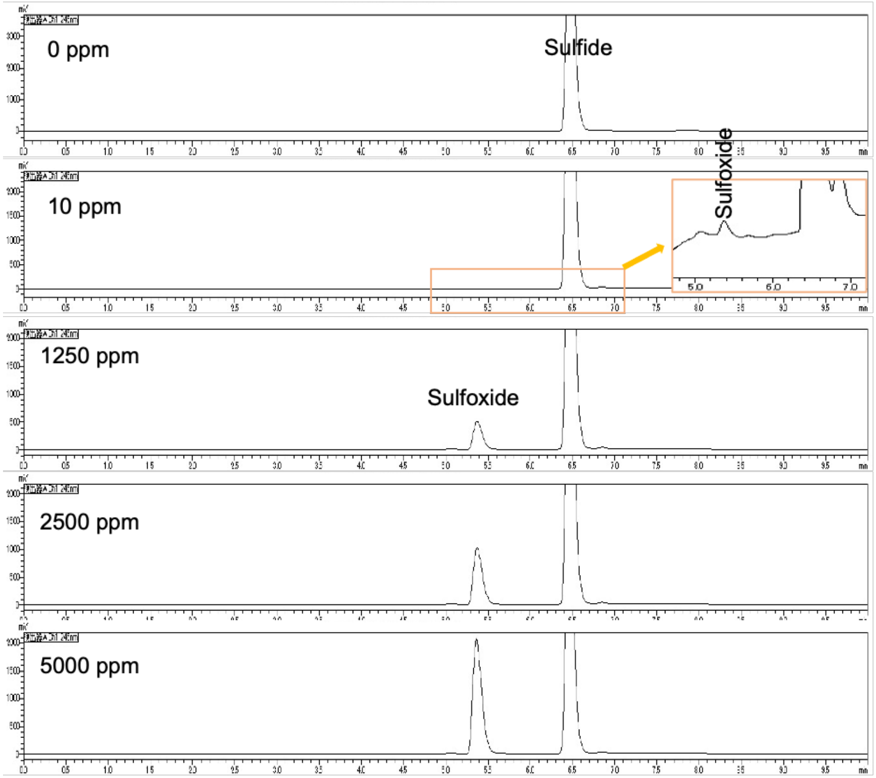

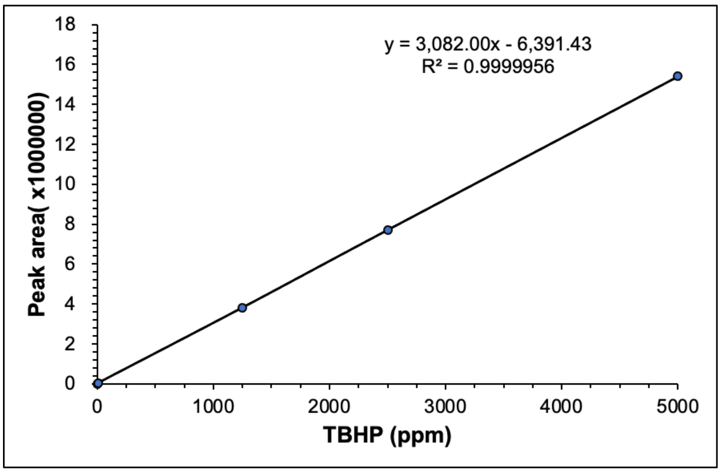

Fig. S1 HPLC chart of benzyl phenyl sulfoxide obtained by oxidizing benzyl phenyl sulfide with *tert*-butyl hydroperoxide (TBHP) and a calibration curve determined from the HPLC UV area

HPLC column: Waters XBridge® oligonucleotide BEH C18 column (4.6×50 mm), Eluent A: H_2O/MeCN/TEA=95/5/0.05, Eluent B: H_2O/MeCN/TEA=5/95/0.05, Flow Rate: 1.0 mL/min, Temperature: 40 °C, Gradient: Eluent B 0%–100% (3 min), Eluent B 100% (1.5 min), Eluent B 100%–0% (0.03 min), Eluent B 0% (5.47 min), Detect: 245 nm.

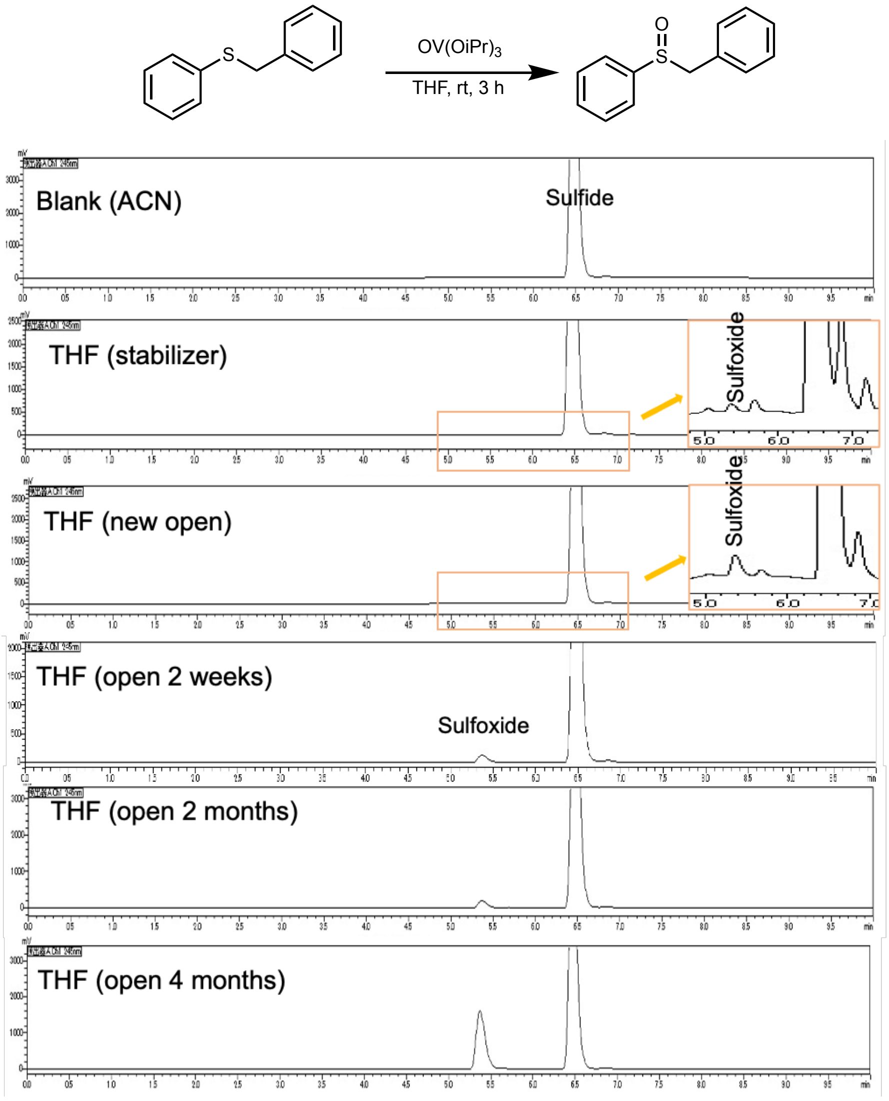

Fig. S2 HPLC chart of benzyl phenyl sulfoxide obtained by oxidizing benzyl phenyl sulfide with THF peroxide

HPLC column: Waters XBridge® oligonucleotide BEH C18 column (4.6×50 mm), Eluent A: H_2O/MeCN/TEA=95/5/0.05, Eluent B: H_2O/MeCN/TEA=5/95/0.05, Flow Rate: 1.0 mL/min, Temperature: 40 °C, Gradient: Eluent B 0%–100% (3 min), Eluent B 100% (1.5 min), Eluent B 100%–0% (0.03 min), Eluent B 0% (5.47 min), Detect: 245 nm.

Table S1 Determination of peroxide concentration in each solvent

Entry	solvent	Peroxide (ppm)	Peroxide (mM)
1	ACN	0	0
2	THF with BHT	7.8	0.0743
3	THF (immediately after opening)	38.1	0.363
4	THF (2 weeks after opening)	301.9	2.87
5	THF (2 months after opening)	483.0	4.60
6	THF (4 months after opening)	3901.2	37.2

2. Relationship between capping conditions and POSP ratio in PS-T5-mer synthesis

Table S2 Peroxide concentration in each solvent and POSP ratio in the crude PS-T5-mer

entry	Solvent conditions	Peroxide (mM)	POPSs (%)
1	ACN	0	1.36
2	THF with BHT	0.0743	1.02
3	THF	0.363	2.21
4	THF	2.87	8.57
5	THF	4.60	12.14
6	THF	37.2	61.00

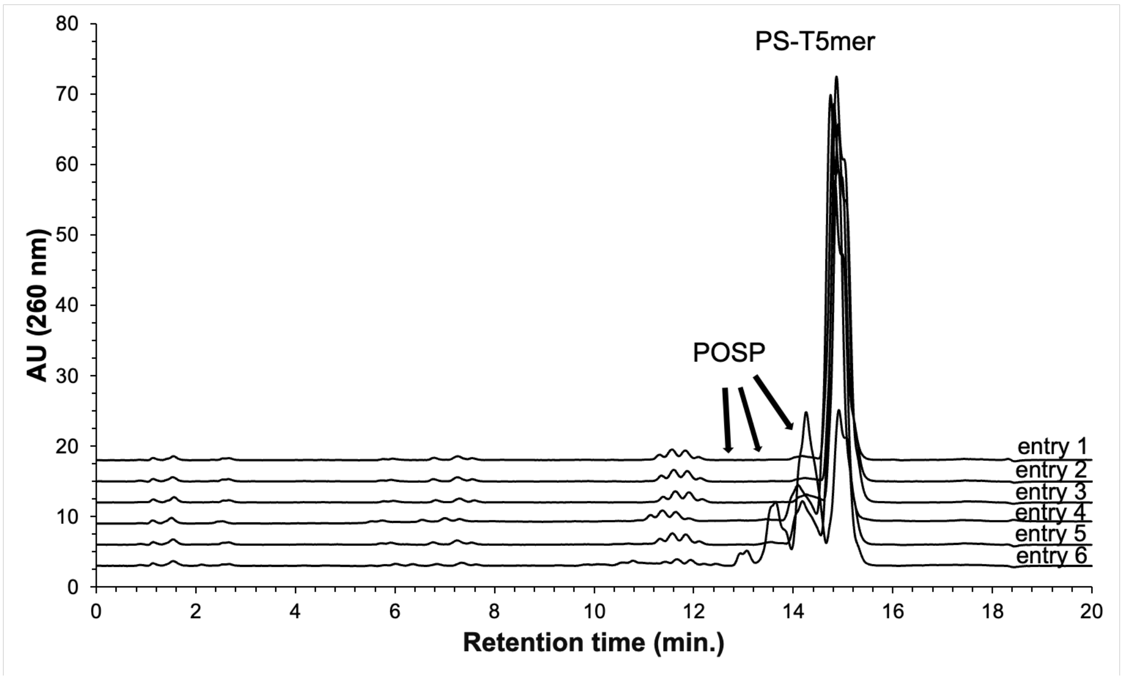

Fig. S3 HPLC charts of crude PS-T5-mer using solvent with adjusted peroxide concentration instead of capping reagent (THF peroxide concentration is shown in Table S2, entries 1–6).

HPLC column: Waters XBridge® oligonucleotide BEH C18 column (4.6×50 mm), Eluent A: H_2O/HFIP/TEA=100/1/0.1, Eluent B: CH_3OH, Flow Rate: 1.0 mL/min, Temperature: 50 °C, Gradient: CH_3OH 0%–15% (20 min).

3. Impurities contained in crude PS-T5-mer synthesised using dT phosphoramidite in THF

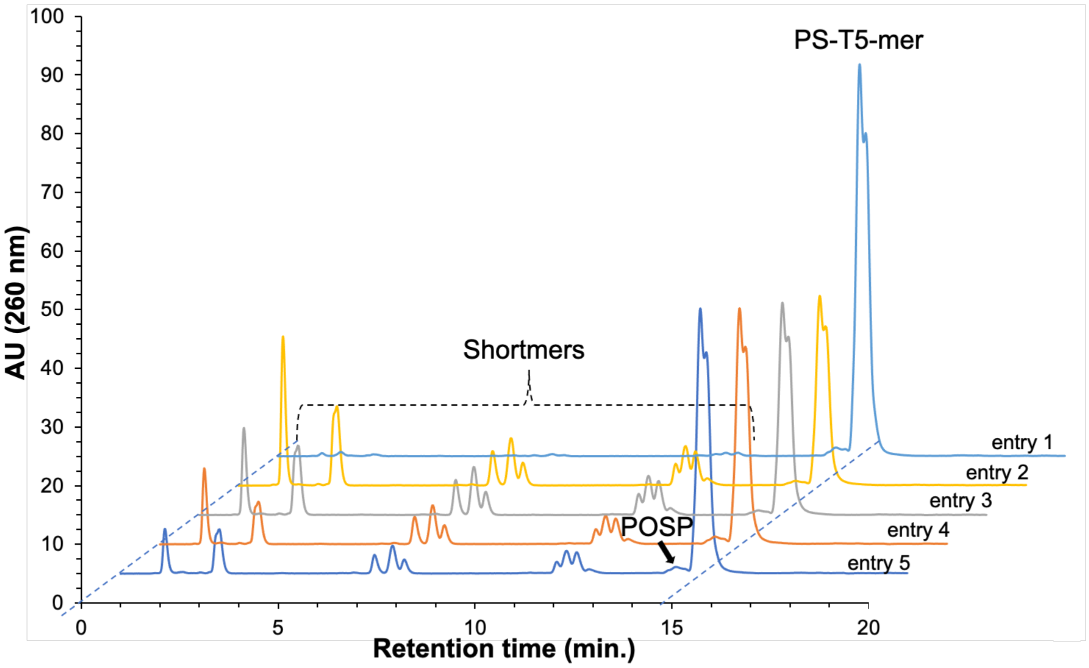

Fig. S4 HPLC charts of PS-T5-mer synthesised using solution of thymidine phosphoramidite with THF peroxide (Solvents are shown in Table 2 and Table S3).

HPLC column: Waters XBridge® oligonucleotide BEH C18 column (4.6×50 mm), Eluent A: H_2O/HFIP/TEA=100/1/0.1, Eluent B: CH_3OH, Flow Rate: 1.0 mL/min, Temperature: 50 °C, Gradient: CH_3OH 0%–15% (20 min).

Table S3 Impurities in crude PS-T5-mer synthesised under different solvents

Entry	Solvents	THF peroxide (mM)	Peak area (%)					
			T1-mer	PS-T2-mer	PS-T3-mer	PS-T4-mer	POSP	PS-T5-mer
1	MeCN	0	0.33	0.39	0.57	1.40	2.44	94.87
2	THF/ MeCN (1/3)	< 0.1	13.51	12.14	12.87	13.13	1.17	47.18
3	THF with BHT/ MeCN (1/3)	4.6	8.02	10.92	13.04	13.32	1.24	53.46
4	THF	< 0.1	7.35	7.00	10.98	10.20	2.13	62.34
5	THF with BHT	4.6	4.43	7.34	7.84	8.33	1.82	70.24

4. Oxidation of thymidine-phophoramidite by THF peroxide

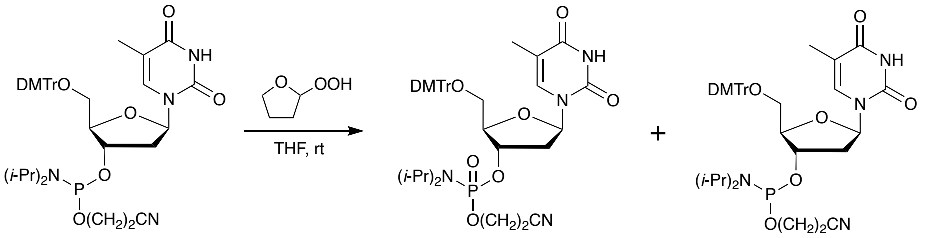

Thymidine-phosphoramidite was dissolved in THF (with and without BHT) and adjusted to a final concentration of 0.067 M. A portion of the reaction solution is withdrawn after 5, 30, 60, and 120 minutes, and THF was removed *in vacuo*. The obtained crude residue was dissolved in methanol and the oxidation ratio was evaluated by LC/MS measurement. The amount of peroxide contained in THF with BHT is approximately 10 ppm. The amount of peroxide contained in THF without BHT is approximately 1336 ppm.

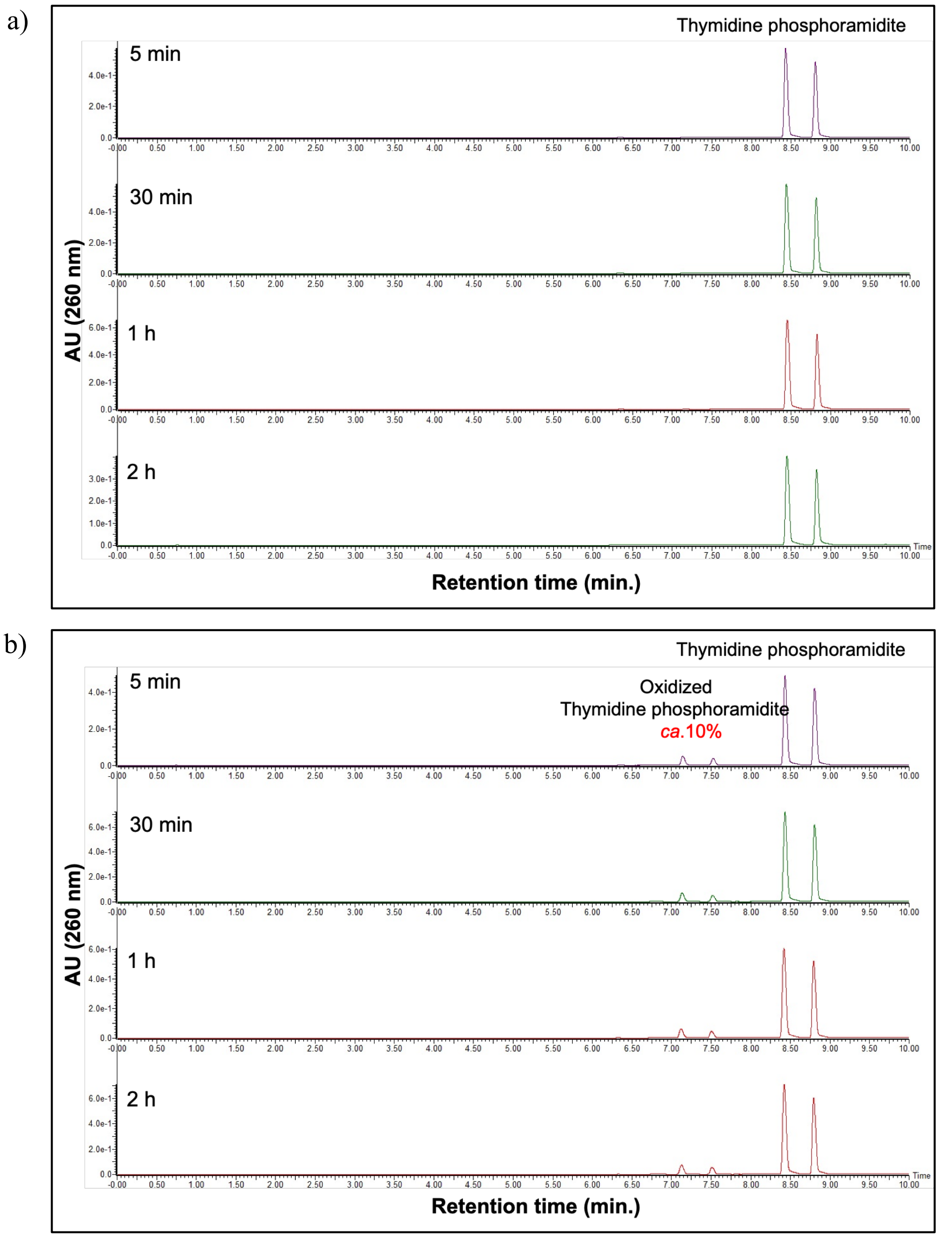

Fig. S5 HPLC chart of thymidine phosphoramidite after treatment with THF a) containing BHT and b) without BHT

LC column: Waters XBridge® oligonucleotide BEH C18 column (2.1×100 mm), Eluent A: H_2O/HFIP/TEA=100/1/0.1, Eluent B: CH_3OH, Flow Rate: 0.3 mL/min, Temperature: 50 °C, Gradient: CH_3OH 60%–100% (10 min).

5. Structure of nusinersen

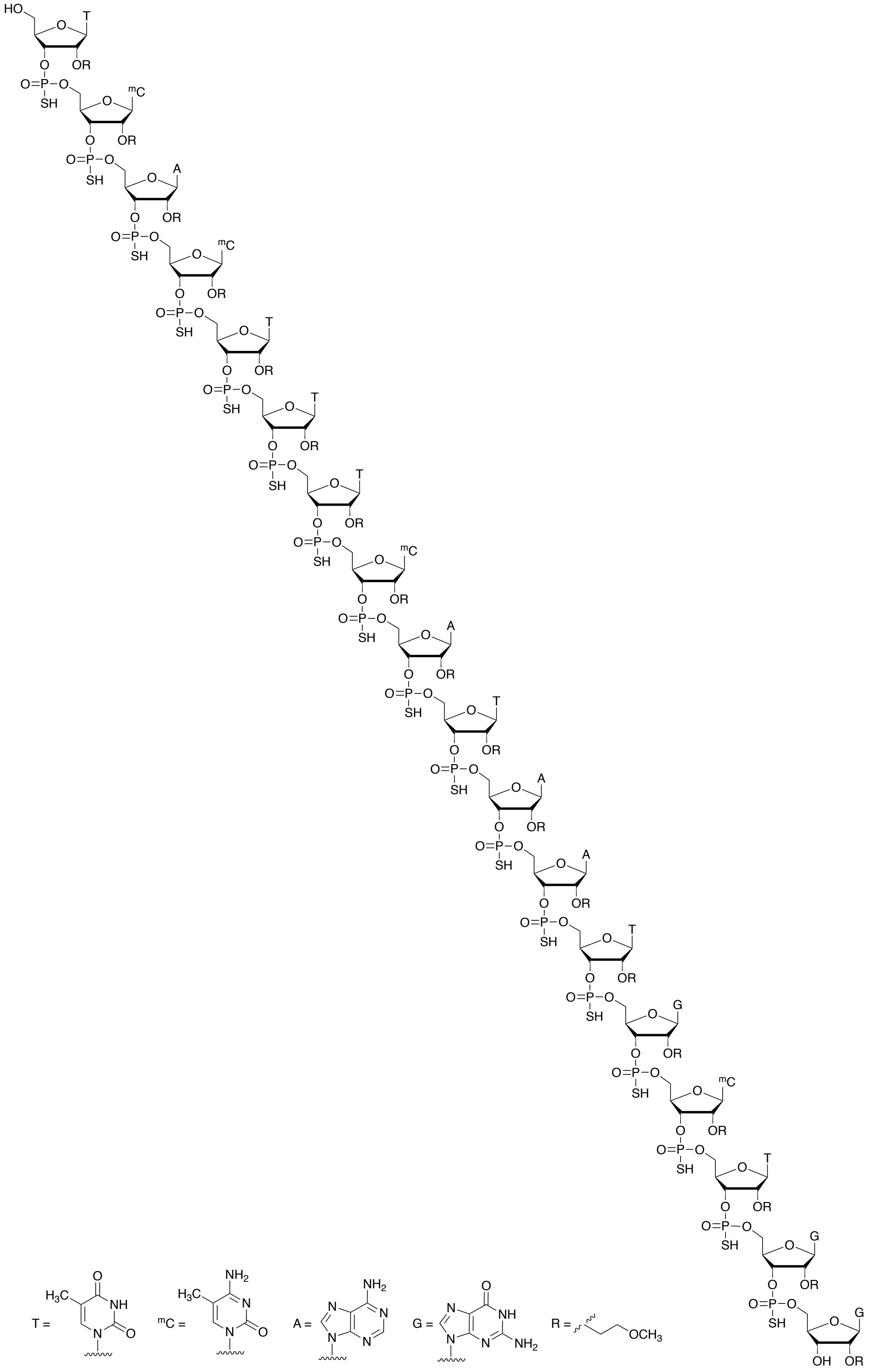

Fig. S6 Nusinersen

6. POSP ratio contained in crude oligonucleotides synthesised by acetyl capping method

1) PS-T5-mer (HPLC)

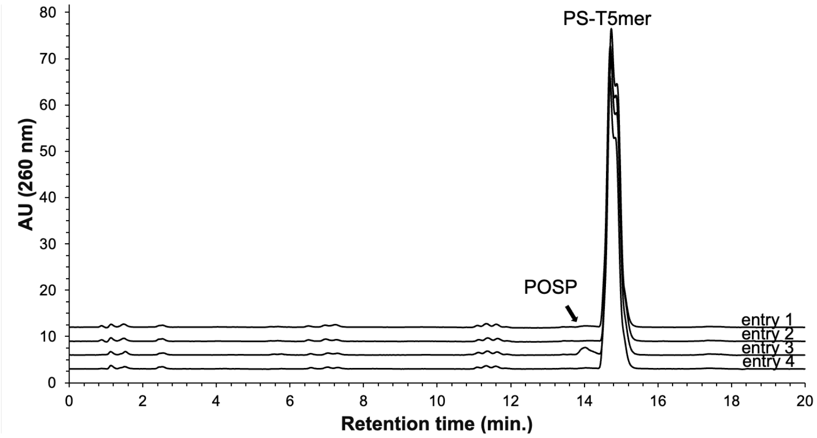

Fig. S7 HPLC charts of PS-T5-mer synthesised by acetyl capping method (Capping reagents are shown in Table 3, Entries 1–4.)

HPLC column: Waters XBridge® oligonucleotide BEH C18 column (4.6×50 mm), Eluent A: H_2O/HFIP/TEA=100/1/0.1, Eluent B: CH_3OH, Flow Rate: 1.0 mL/min, Temperature: 50 °C, Gradient: CH_3OH 0%–15% (20 min).

2) Nusinersen (LC/MS)

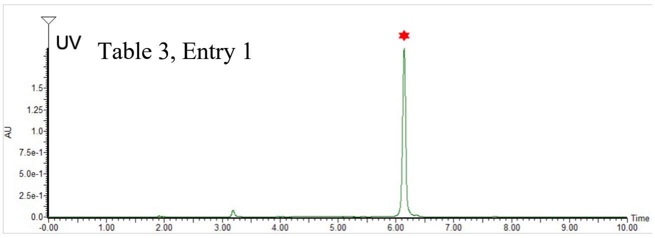

	Average value of Deconvolution Mass (Da)	Intensity	%Relative	%Total
Nusinersen	7126.7	1.51E+008	100	90.85
Nusinersen+CE	7179.5	1.03E+007	6.77	6.15
POSP (1)	7110.9	4.99E+006	3.29	2.99

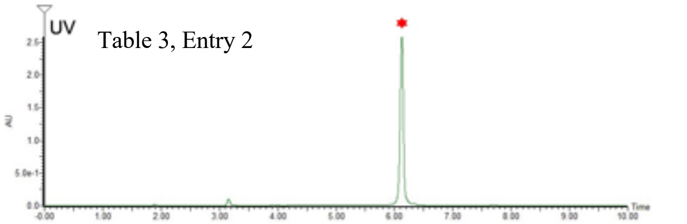

	Average value of Deconvolution Mass (Da)	Intensity	%Relative	%Total
Nusinersen	7126.7	1.65E+008	100	92.97
Nusinersen+CE	7179.5	9.58E+006	5.81	5.40
POSP (1)	7110.6	2.88E+006	1.75	1.63

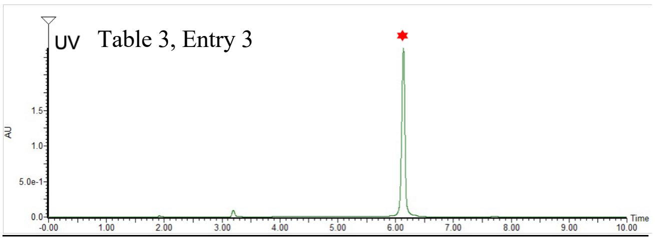

	Average value of Deconvolution Mass (Da)	Intensity	%Relative	%Total
Nusinersen	7126.7	1.40E+008	100	78.73
POSP (1)	7110.7	2.67E+007	19.04	14.99
Nusinersen+CE	7179.5	8.73E+006	6.24	4.91
POSP (2)	7094.0	2.43E+006	1.73	1.37

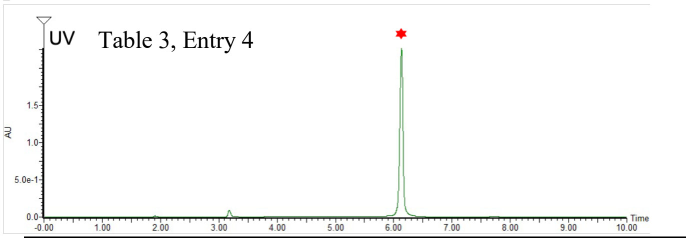

	Average value of Deconvolution Mass (Da)	Intensity	%Relative	%Total
Nusinersen	7126.7	1.60E+008	100	92.32
Nusinersen+CE	7179.5	1.01E+007	6.30	5.81
POSP (1)	7111.0	3.23E+006	2.02	1.87

POSP (1): Numbers in parentheses indicate the number of desulphurised PS bonds. "CE" means that N3 of thymine is 2-cyanoethylated.

Fig. S8 LC charts of nusinersen synthesised by acetyl capping method and POSP ratios contained in crude nusinersen according to deconvolution mass intensity (Capping reagents are shown in Table 3, Entries 1–4.)

LC column: Waters XBridge® oligonucleotide BEH C18 Column 1.7 μm, 2.1×100 mm, Eluent A: H_2O/HFIP/TEA=100/1/0.1, Eluent B: CH_3OH, Flow Rate: 0.3 mL/min, Temperature: 50 °C, Gradient: CH_3OH 5%–70% (10 min).

7. POSP ratio contained in oligonucleotides synthesised by cholesterol capping method

1) PS-T5-mer (HPLC)

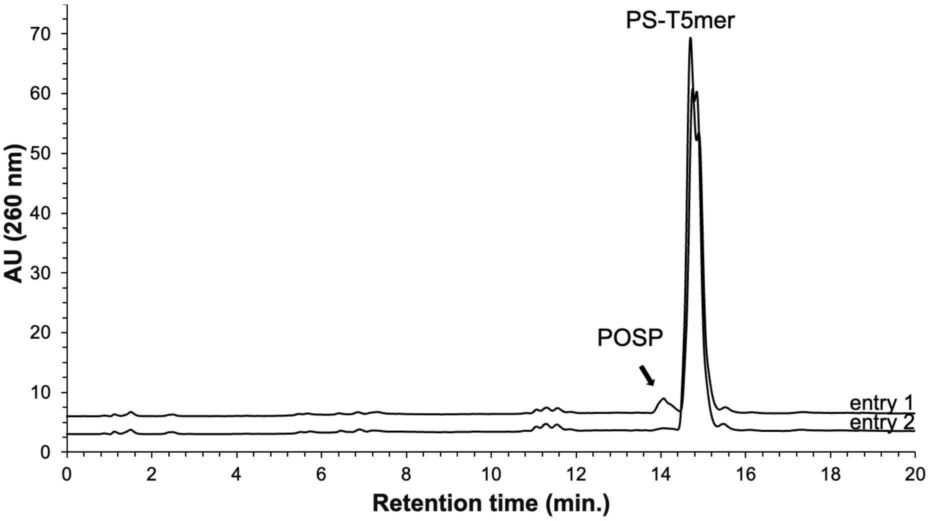

Fig. S9 HPLC charts of oligonucleotides synthesised by cholesterol capping (Table 4, Entry1).

HPLC column: Waters XBridge® oligonucleotide BEH C18 column (4.6x50 mm), Eluent A: H_2O/HFIP/TEA=100/1/0.1, Eluent B: CH_3OH, Flow Rate: 1.0 mL/min, Temperature: 50 °C, Gradient: CH_3OH 0%–15% (20 min).

2) Nusinersen (LC/MS)

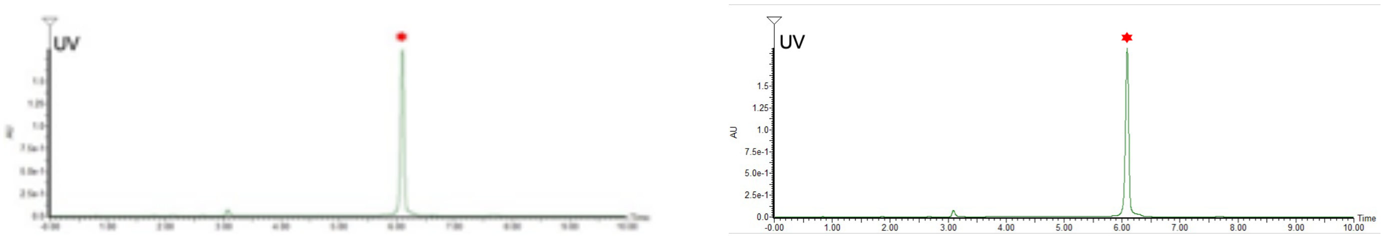

	Average value of Deconvolution Mass (Da)	Intensity	%Relative	%Total
Nusinersen	7126.8	1.78E+008	100	82.20
POSP (1)	7110.5	3.50E+007	19.69	16.19
POSP (2)	7094.1	3.47E+006	1.95	1.61

	Average value of Deconvolution Mass (Da)	Intensity	%Relative	%Total
Nusinersen	7126.8	2.22E+008	100	95.97
POSP (1)	7110.8	9.33E+006	4.20	4.03

POSP (1): Numbers in parentheses indicate the number of desulphurised PS bonds.

Fig. S10 LC charts of nusinersen synthesised by acetyl capping method and POSP ratios contained in crude nusinersen according to deconvolution mass intensity (Capping reagents are shown in Table 4, Entries 1 and 2.)

LC column: Waters XBridge® oligonucleotide BEH C18 Column 1.7 µm, 2.1×100 mm, Eluent A: H_2O/HFIP/TEA=100/1/0.1, Eluent B: CH_3OH, Flow Rate: 0.3 mL/min, Temperature: 50 °C, Gradient: CH_3OH 5%–70% (10 min).

8. LC-MS data of oligonucleotides

LC column: Waters XBridge® oligonucleotide BEH C18 Column 1.7 µm, 2.1 ×100 mm
Eluent A: H_2O/HFIP/TEA=100/1/0.1, Eluent B: CH_3OH
Flow Rate: 0.3 mL/min, Temperature: 50 °C, Gradient: CH_3OH 5%-15% (10 min).

1) LC (UV) and MS charts of entry 1-8 (PS-T5-mer) in Table 1

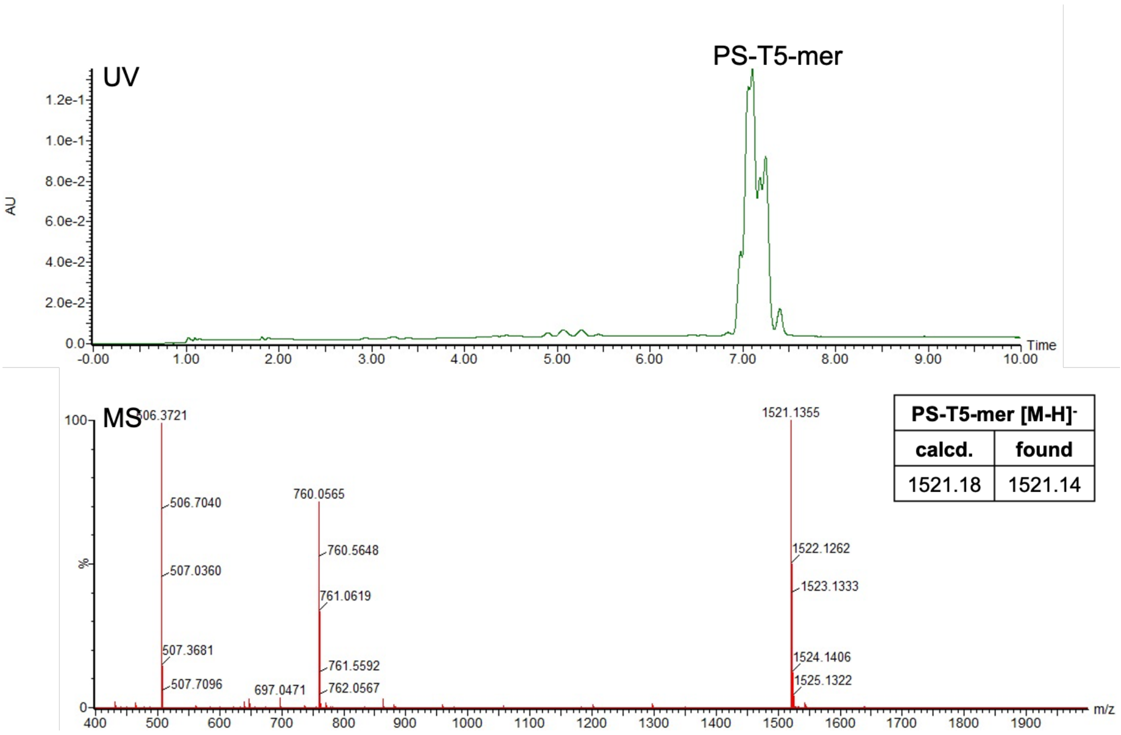

Fig. S11 LC (UV) and MS charts of crude PS-T5-mer synthesised without capping (Table 1, entry 1).

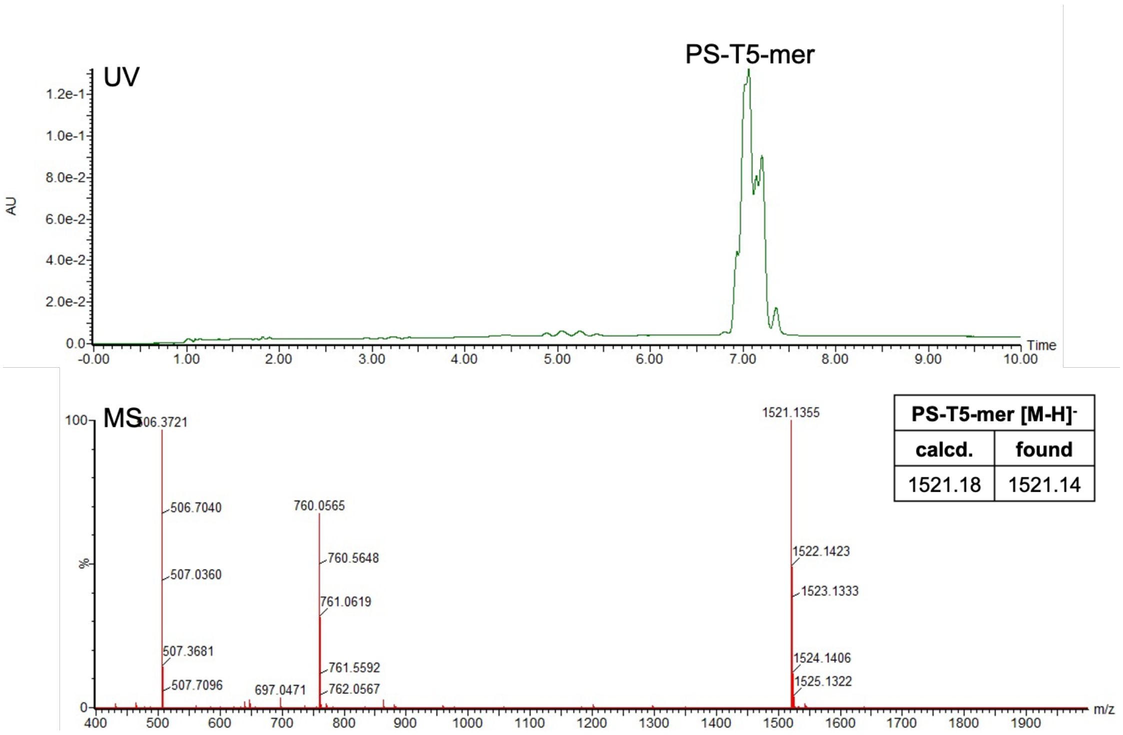

Fig. S12 LC (UV) and MS charts of crude PS-T5-mer synthesised with cholesterol capping after sulphurisation (Table 1, entry 2).

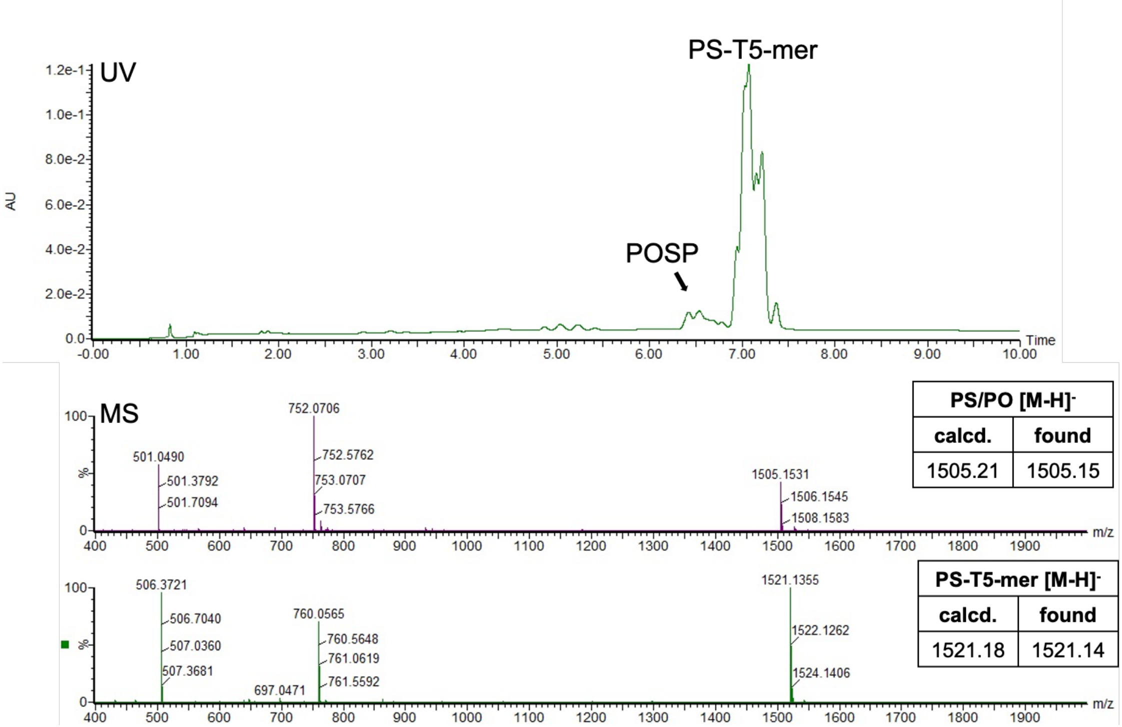

Fig. S13 LC (UV) and MS charts of crude PS-T5-mer synthesised with cholesterol capping before sulphurisation (Table 1, entry 3).

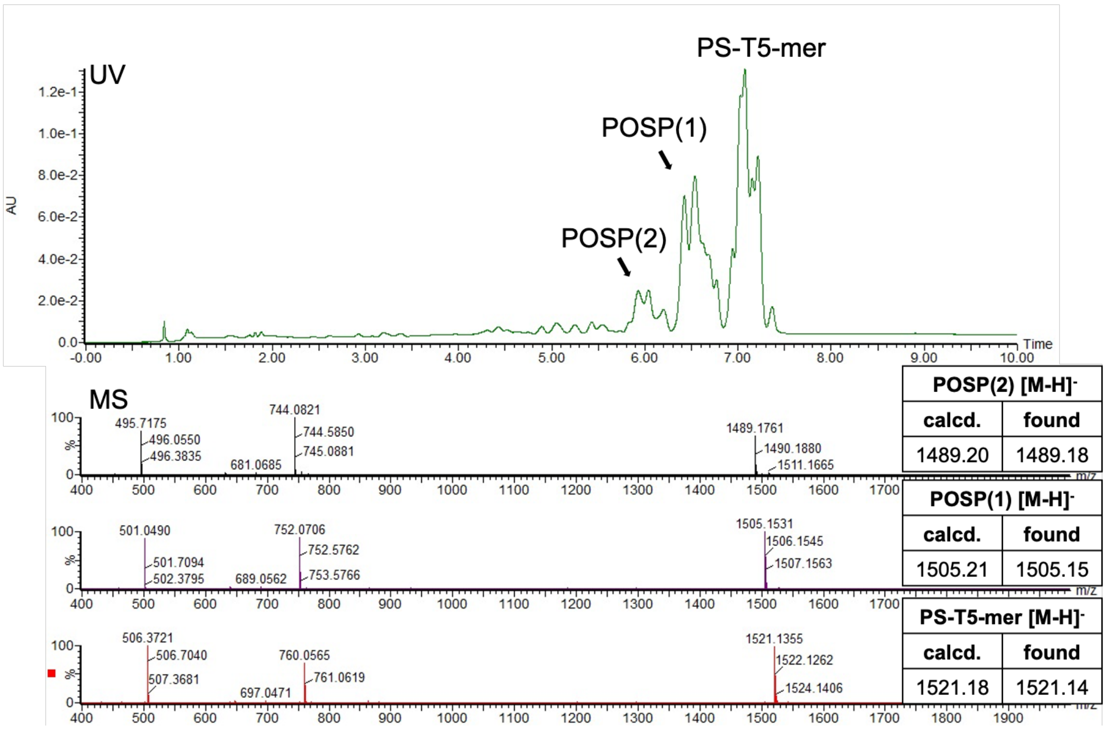

Fig. S14 LC (UV) and MS chart of crude PS-T5-mer synthesised under conditions of flowing THF and ETT instead of cholesterol capping (Table 1, entry 4).

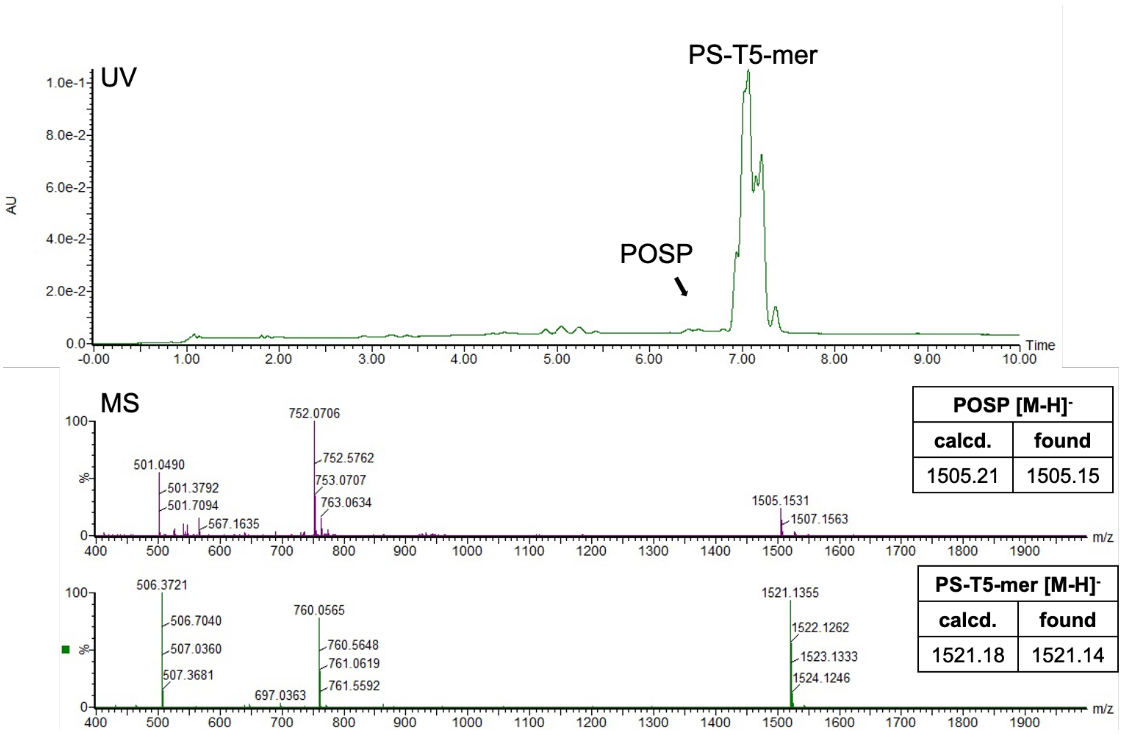

Fig. S15 LC (UV) and MS charts of MS chart of crude PS-T5-mer synthesised under conditions of flowing MeCN and ETT instead of cholesterol capping (Table 1, entry 5).

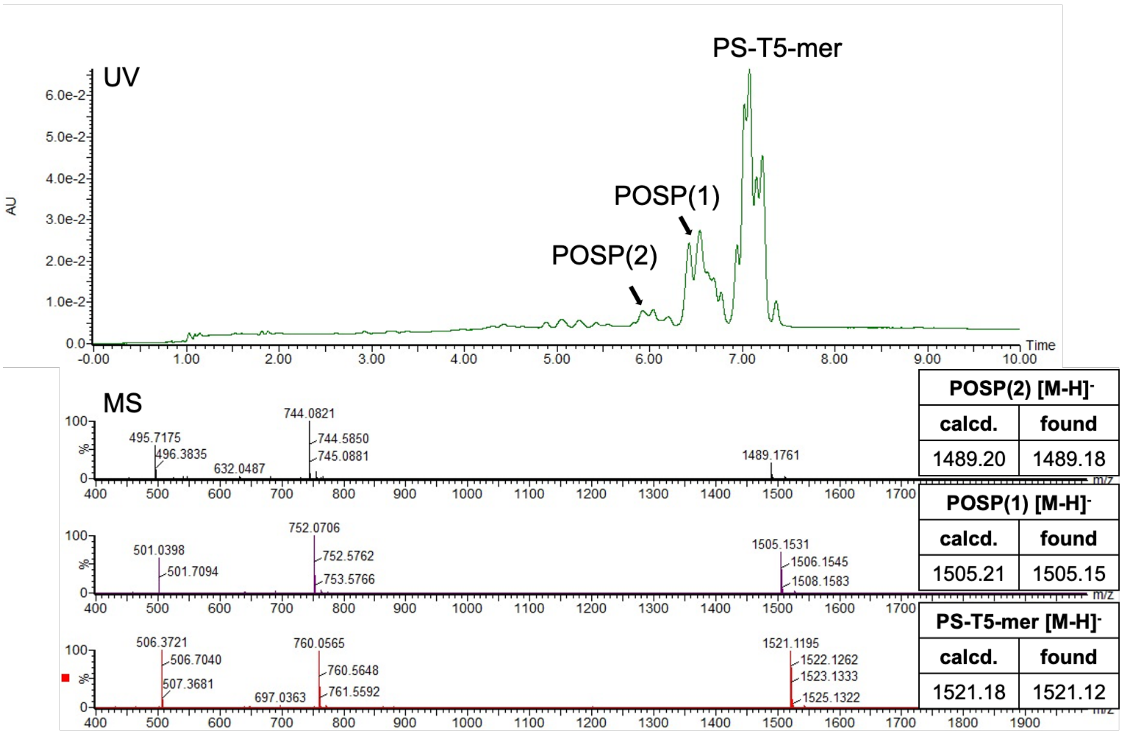

Fig. S16 LC (UV) and MS charts of MS chart of crude PS-T5-mer synthesised under conditions of flowing THF (with BHT) only instead of cholesterol capping (Table 1, entry 6).

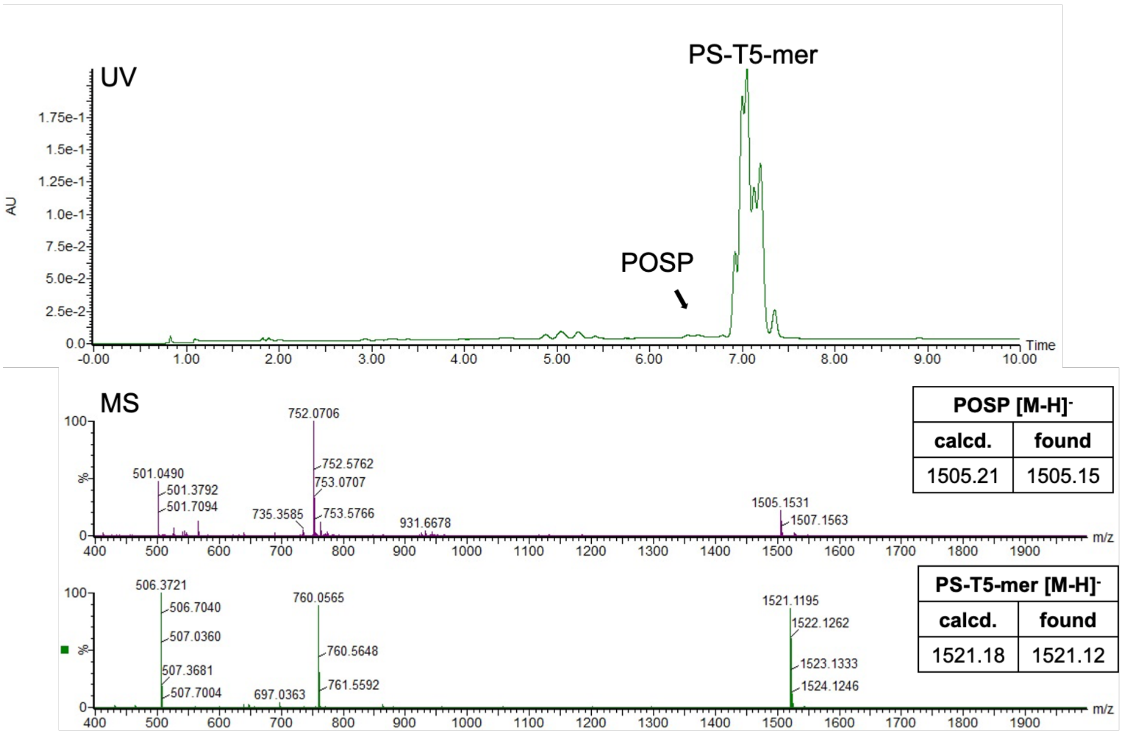

Fig. S17 LC (UV) and MS charts of MS chart of crude PS-T5-mer synthesised under conditions of flowing THF (without BHT) instead of cholesterol capping (Table 1, entry 7).

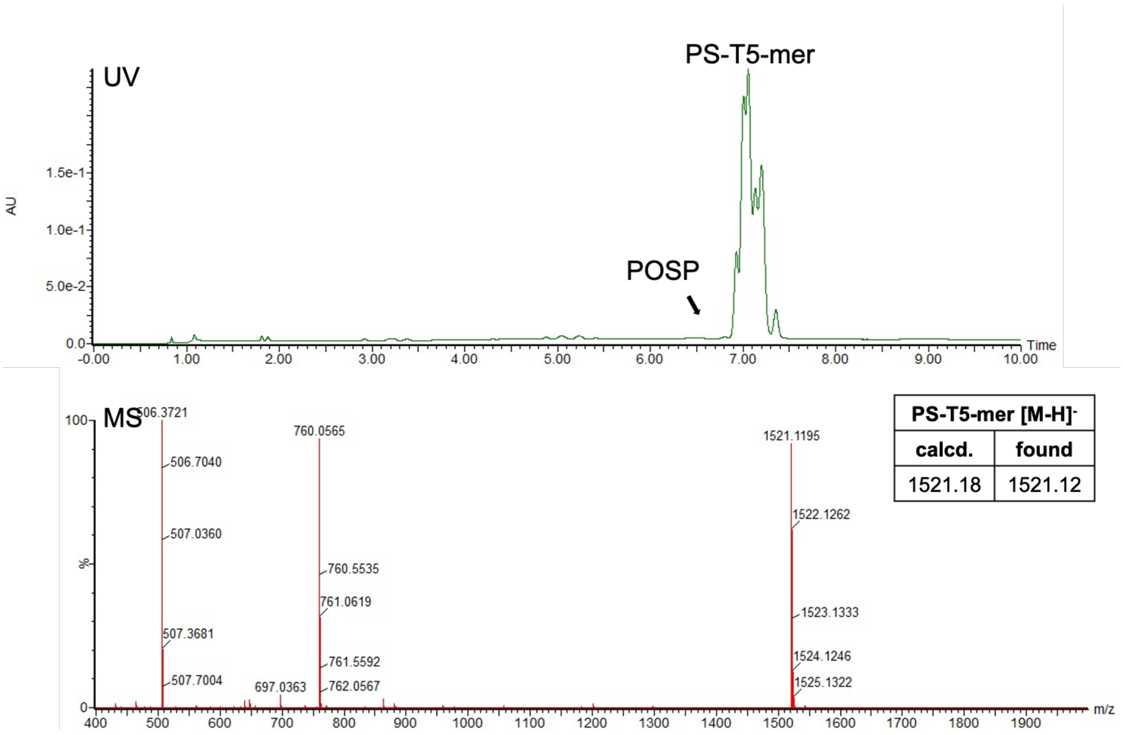

Fig. S18 LC (UV) and MS charts of crude PS-T5-mer synthesised with acetyl capping (Table 1, entry 8).

2) LC (UV) and MS charts of entry 1-5 (PS-T5-mer) in Table 2.

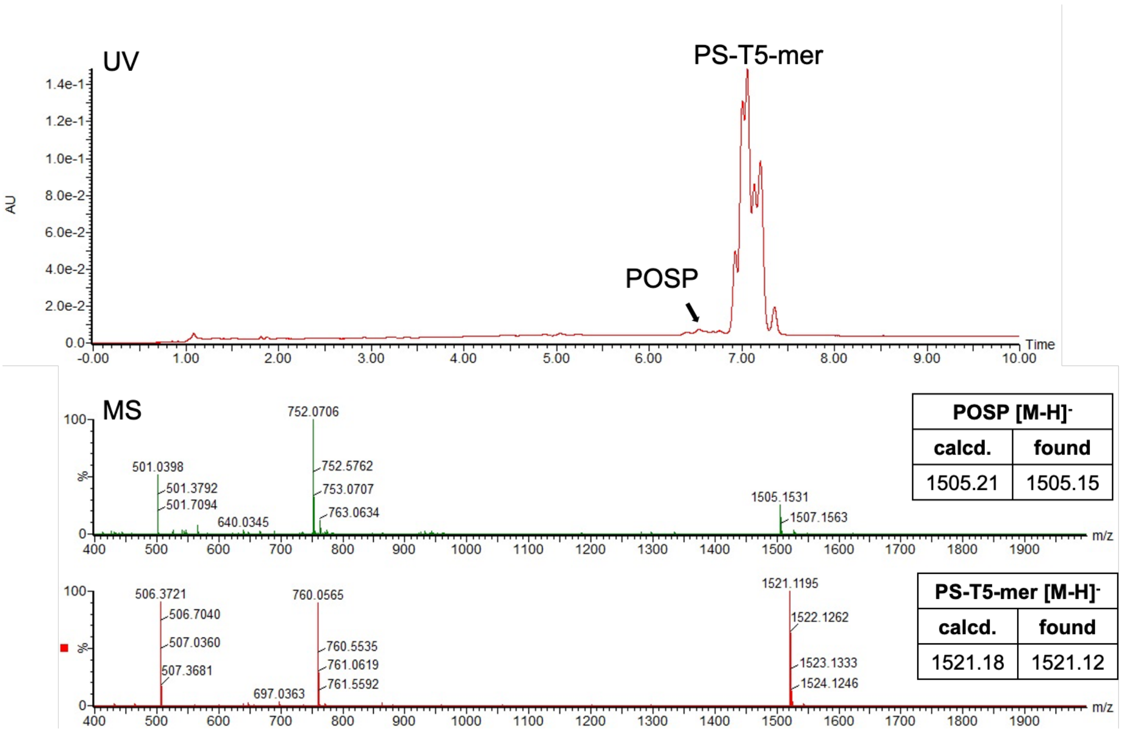

Fig. S19 LC (UV) and MS charts of crude PS-T5-mer synthesised using solution of thymidine phosphoramidite without THF peroxide (Table 2, entry 1).

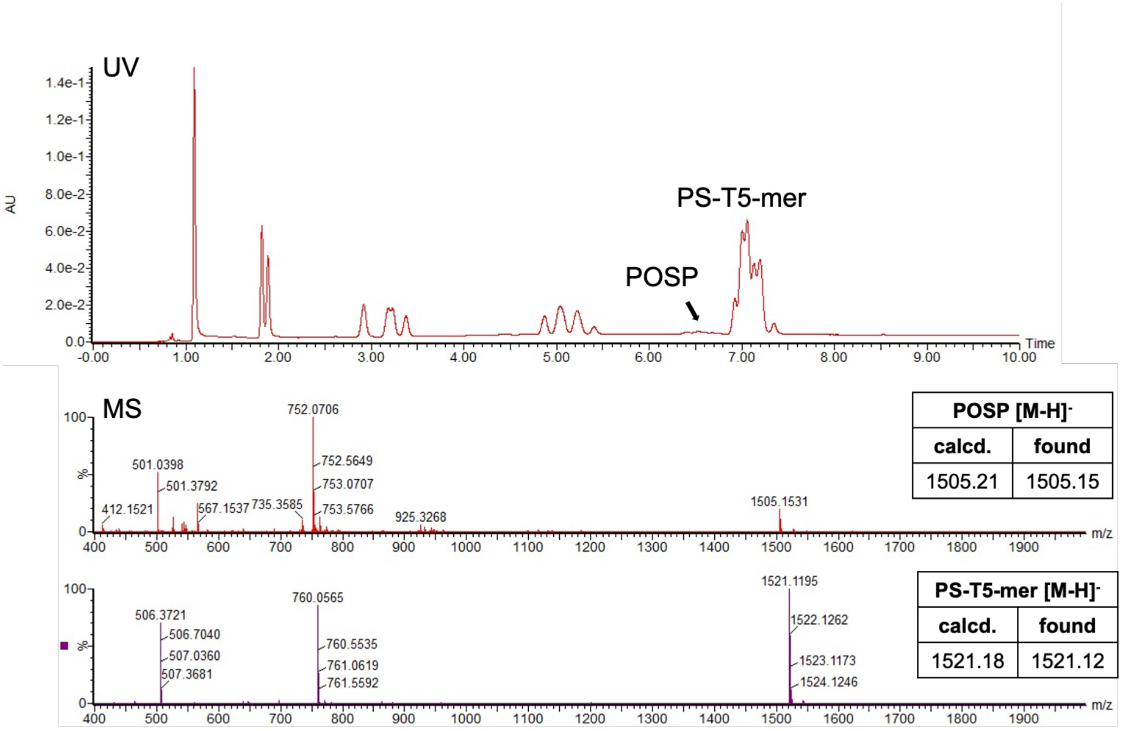

Fig. S20 LC (UV) and MS charts of crude PS-T5-mer synthesised using solution of thymidine phosphoramidite with THF peroxide (Table 2, entry 2).

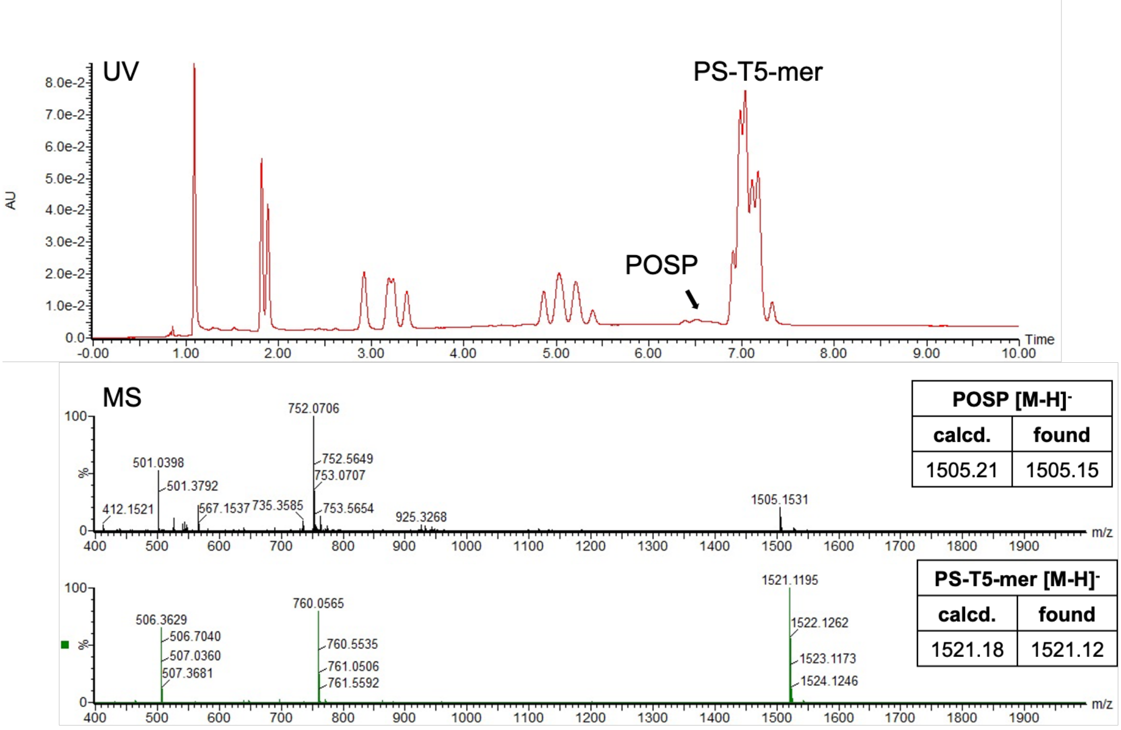

Fig. S21 LC (UV) and MS charts of crude PS-T5-mer synthesised using solution of thymidine phosphoramidite with THF peroxide (Table 2, entry 3).

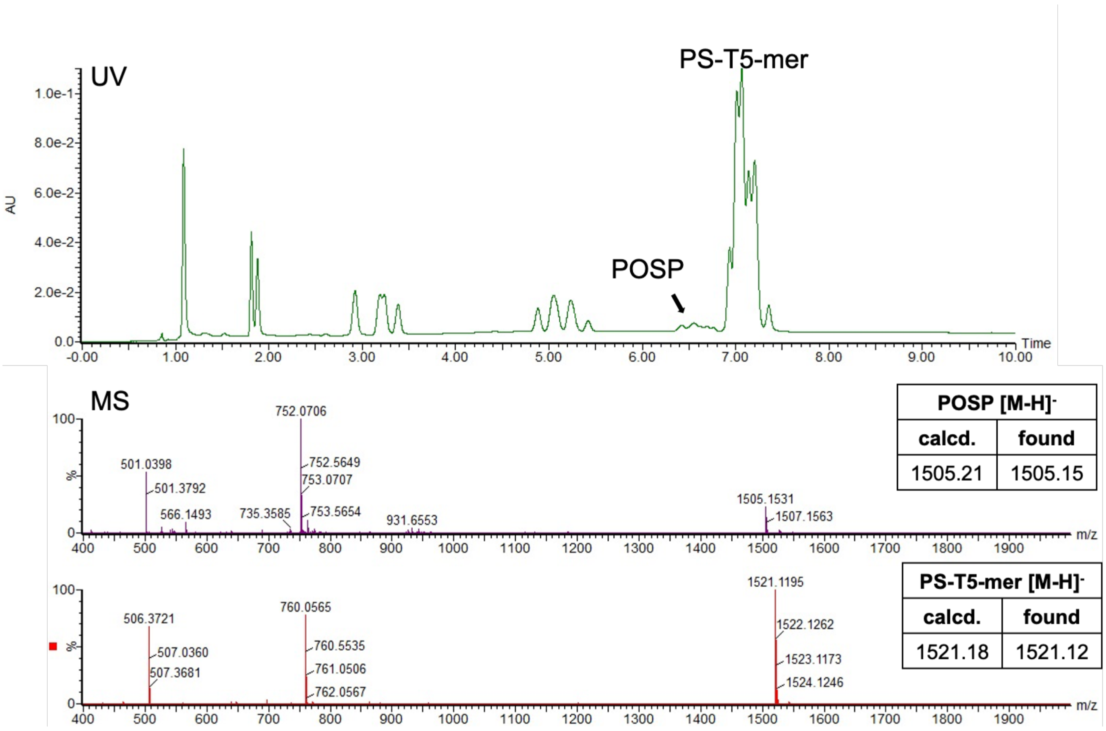

Fig. S22 LC (UV) and MS charts of crude PS-T5-mer synthesised using solution of thymidine phosphoramidite with THF peroxide (Table 2, entry 4).

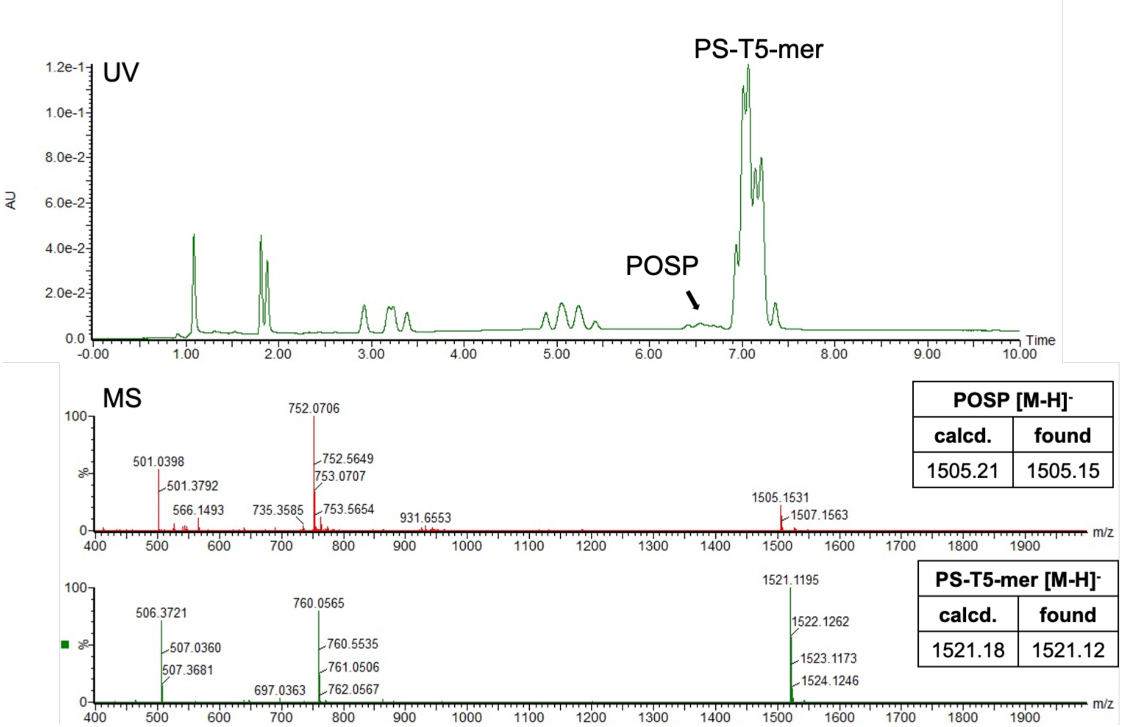

Fig. S23 LC (UV) and MS charts of crude PS-T5-mer synthesised using solution of thymidine phosphoramidite with THF peroxide (Table 2, entry 5).

3) LC (UV) and MS charts of entry 1-6 (PS-T5-mer) in Table. S2

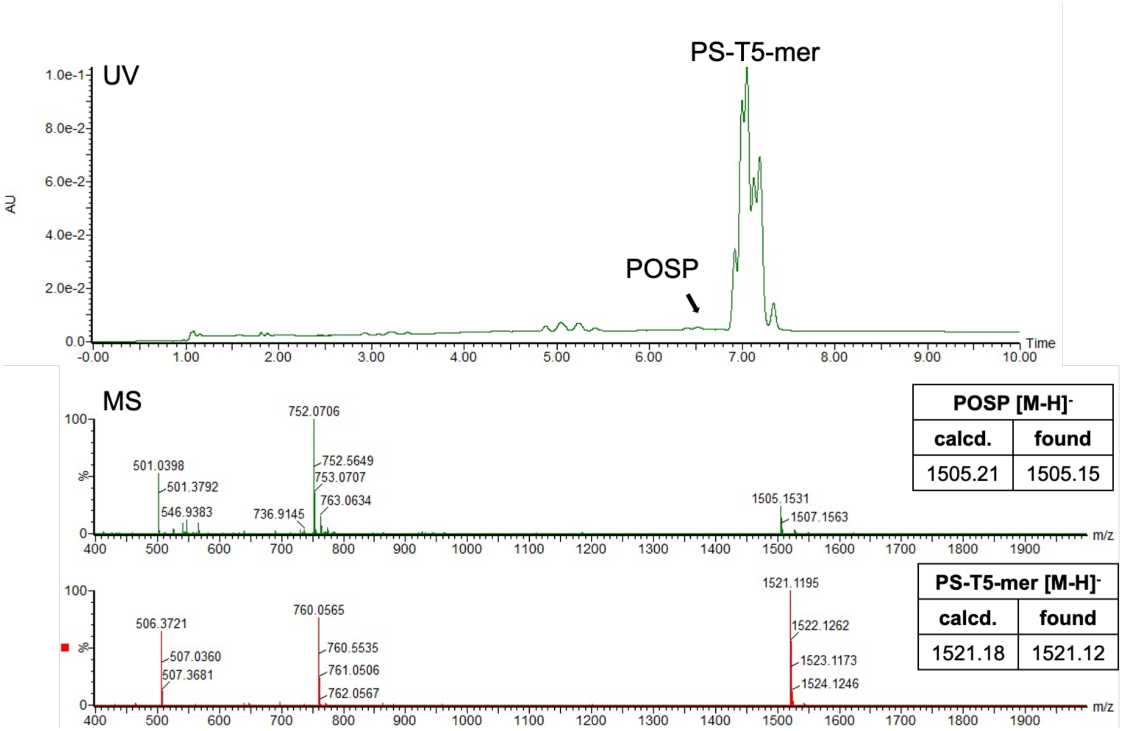

Fig. S24 LC (UV) and MS charts of crude PS-T5-mer synthesised under conditions of flowing MeCN instead of cholesterol capping (Table S2, entry 1).

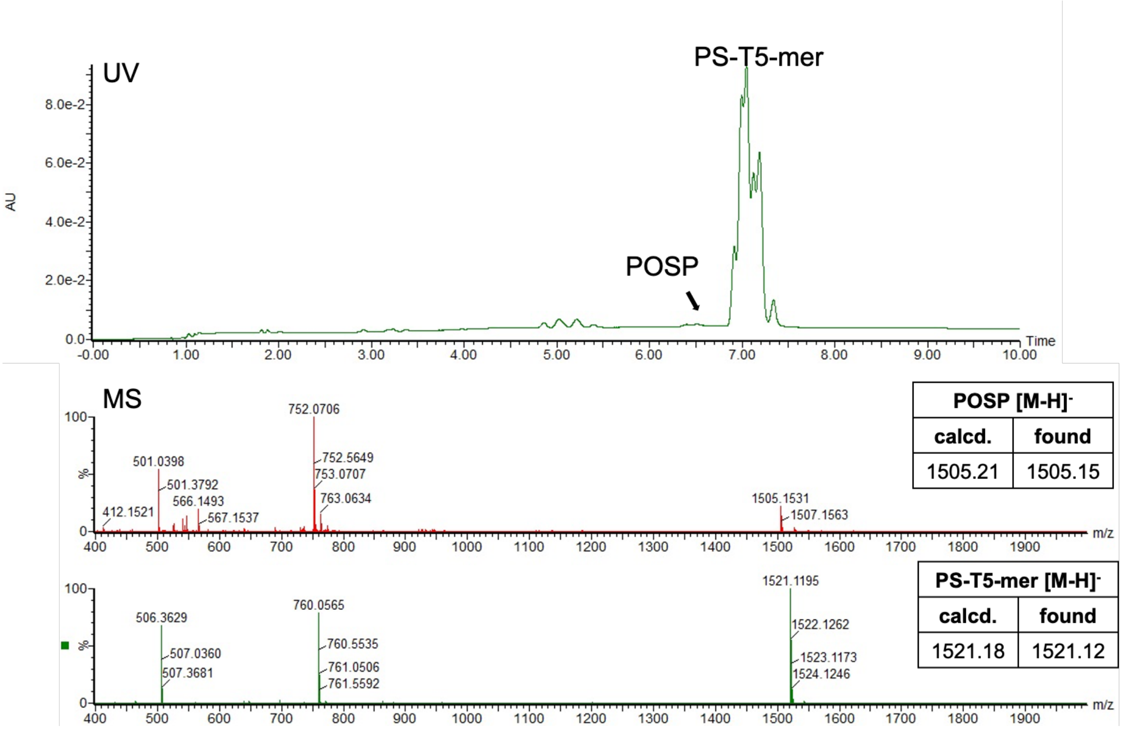

Fig. S25 LC (UV) and MS charts of crude PS-T5-mer synthesised under conditions of flowing THF (with BHT) instead of cholesterol capping (Table S2, entry 2).

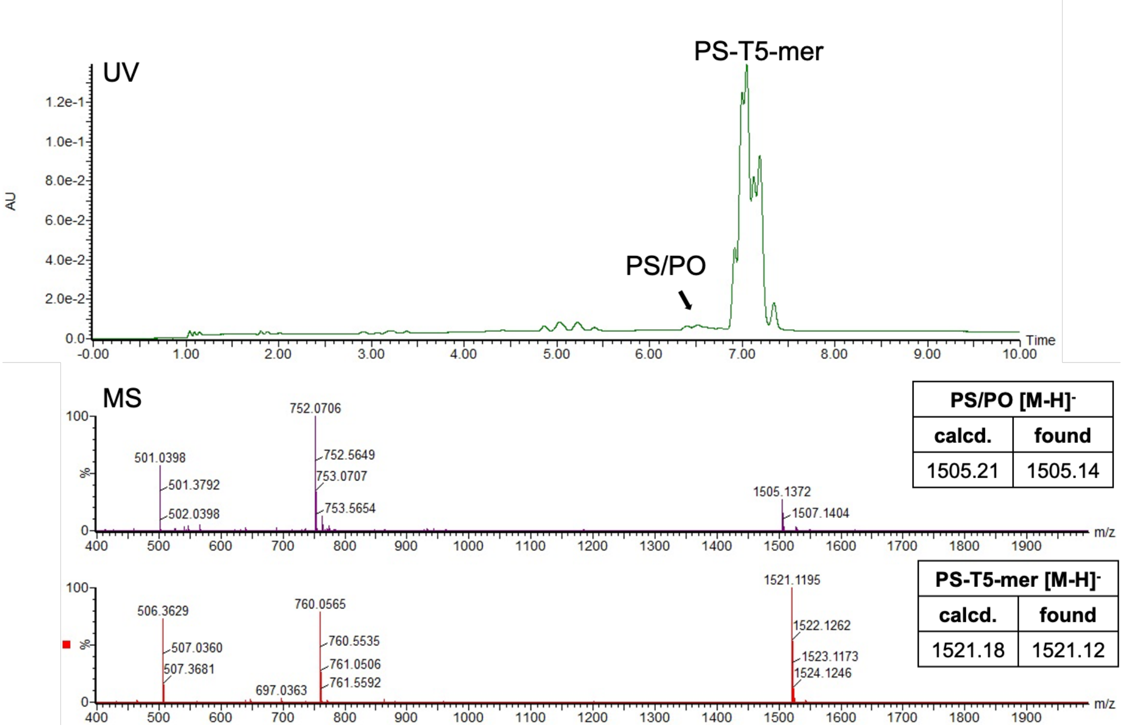

Fig. S26 LC (UV) and MS charts of crude PS-T5-mer synthesised under conditions of flowing THF (without BHT) instead of cholesterol capping (Table S2, entry 3).

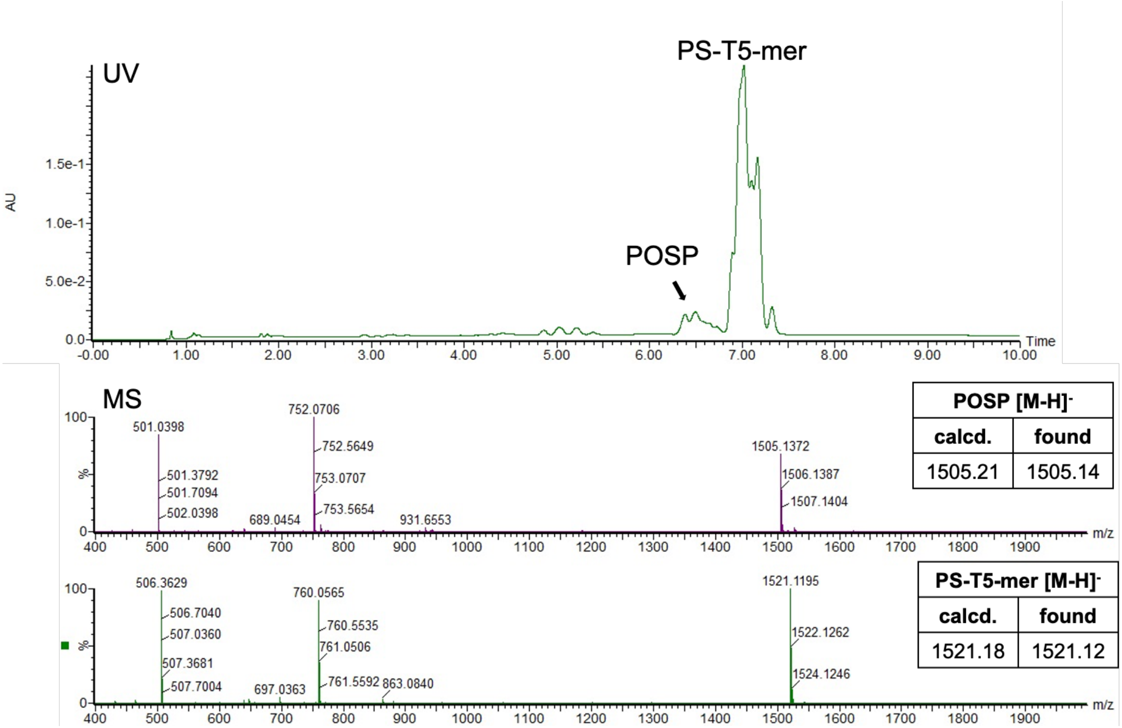

Fig. S27 LC (UV) and MS charts of crude PS-T5-mer synthesised under conditions of flowing THF (without BHT) instead of cholesterol capping (Table S2, entry 4).

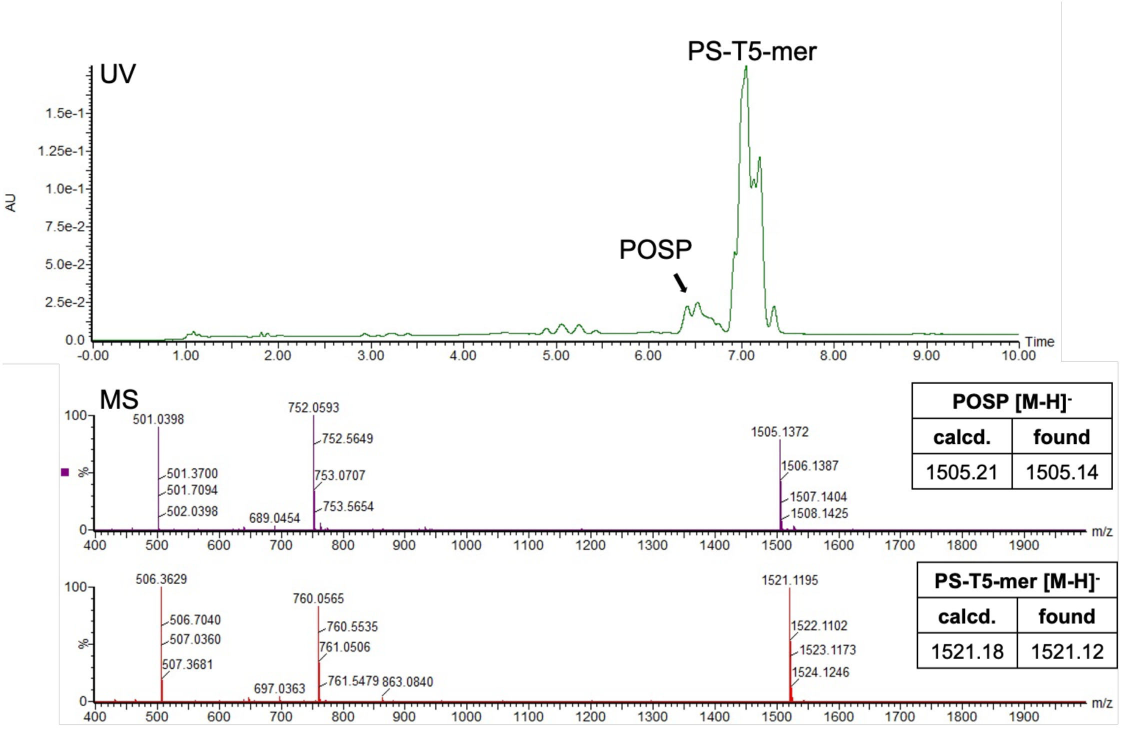

Fig. S28 LC (UV) and MS charts of crude PS-T5-mer synthesised under conditions of flowing THF (without BHT) instead of cholesterol capping (Table S2, entry 5).

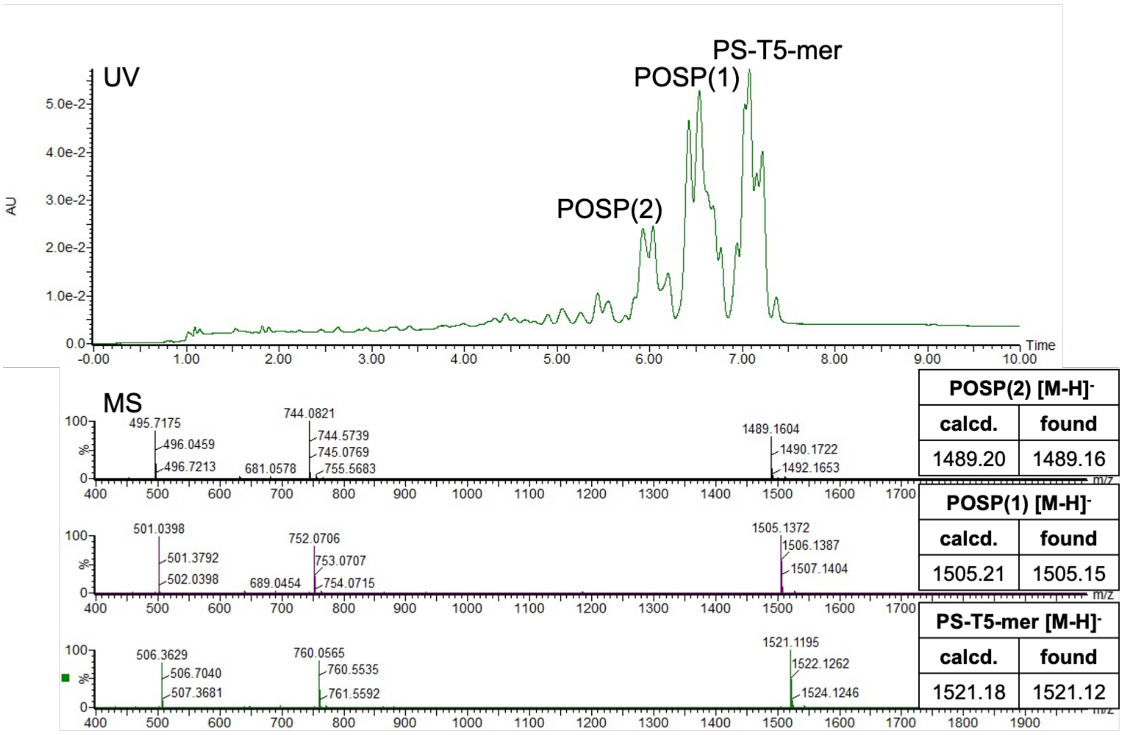

Fig. S29 LC (UV) and MS charts of crude PS-T5-mer synthesised under conditions of flowing THF (without BHT) instead of cholesterol capping (Table S2, entry 6).

4) LC (UV) and MS charts of entry 1-4 (PS-T5-mer) in Table 3

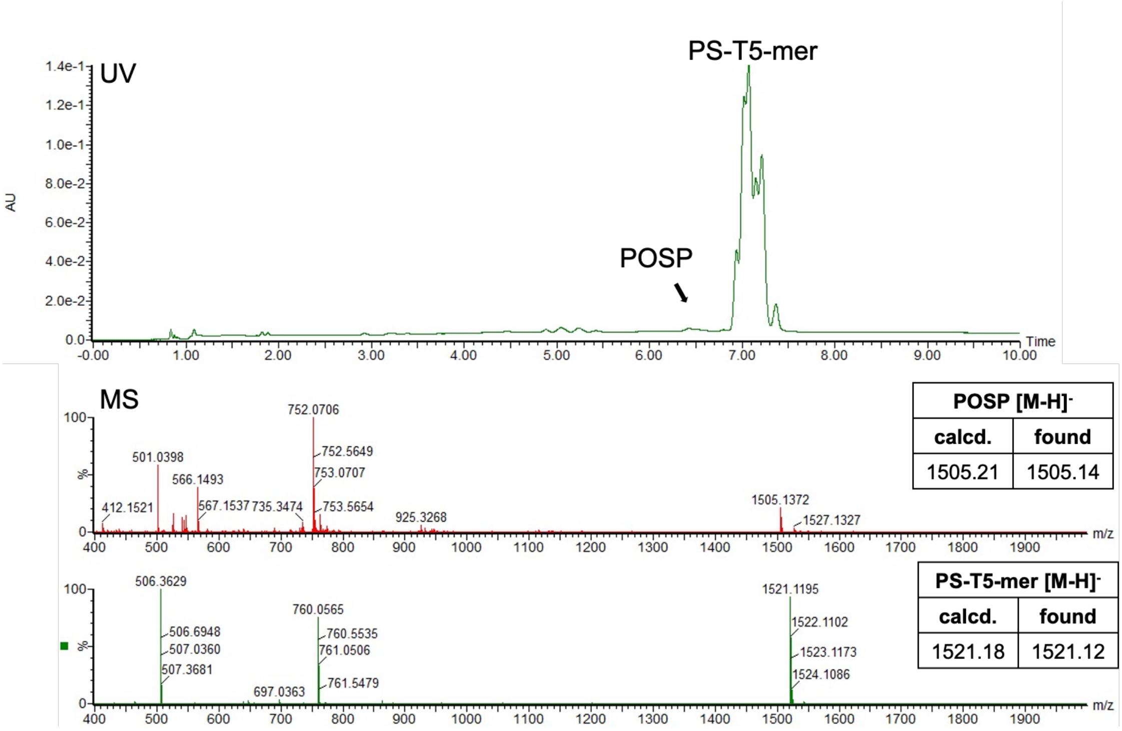

Fig. S30 LC (UV) and MS charts of crude PS-T5-mer synthesised by acetyl capping method (THF with BHT) (Table 3, entry 1).

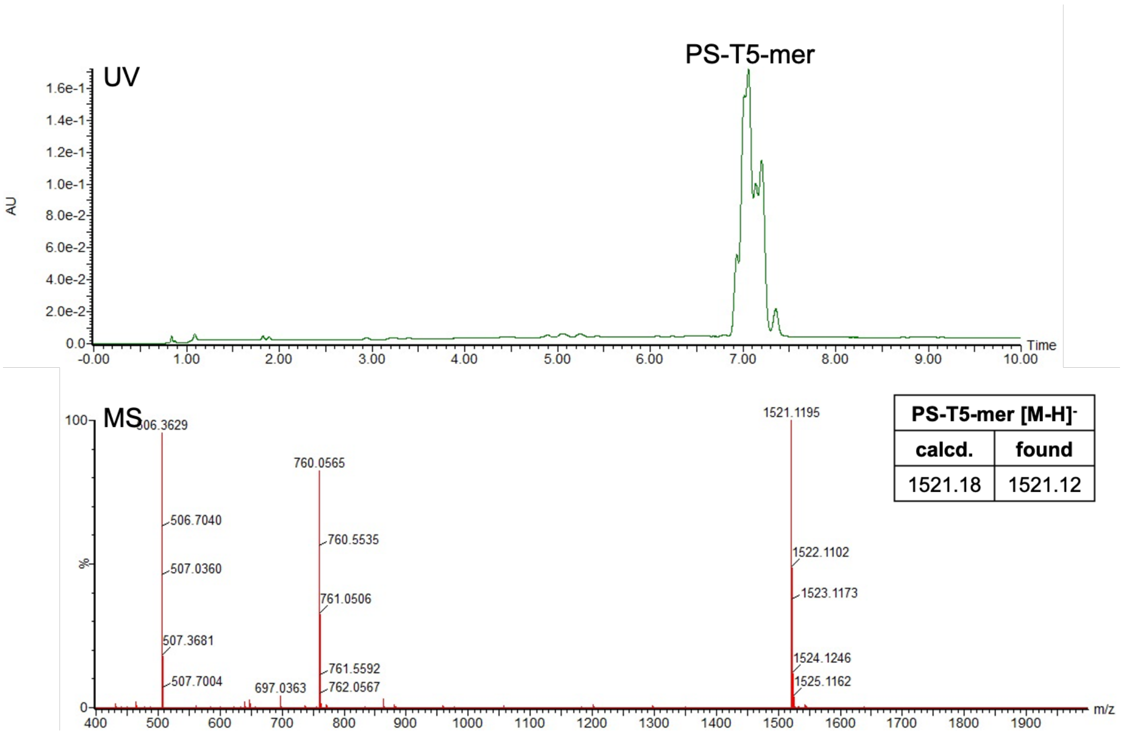

Fig. S31 LC (UV) and MS charts of crude PS-T5-mer synthesised by acetyl capping method (THF with BHT) (Table 3, entry 2).

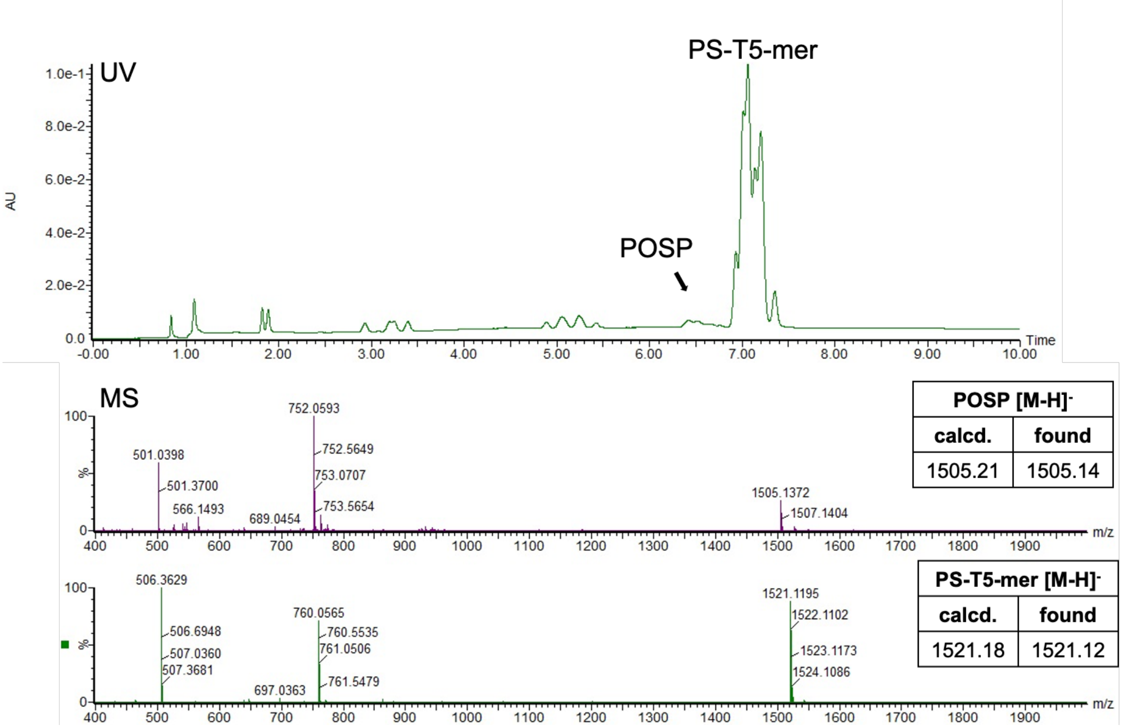

Fig. S32 LC (UV) and MS charts of crude PS-T5-mer synthesised by acetyl capping method (THF without BHT) (Table 3, entry 3).

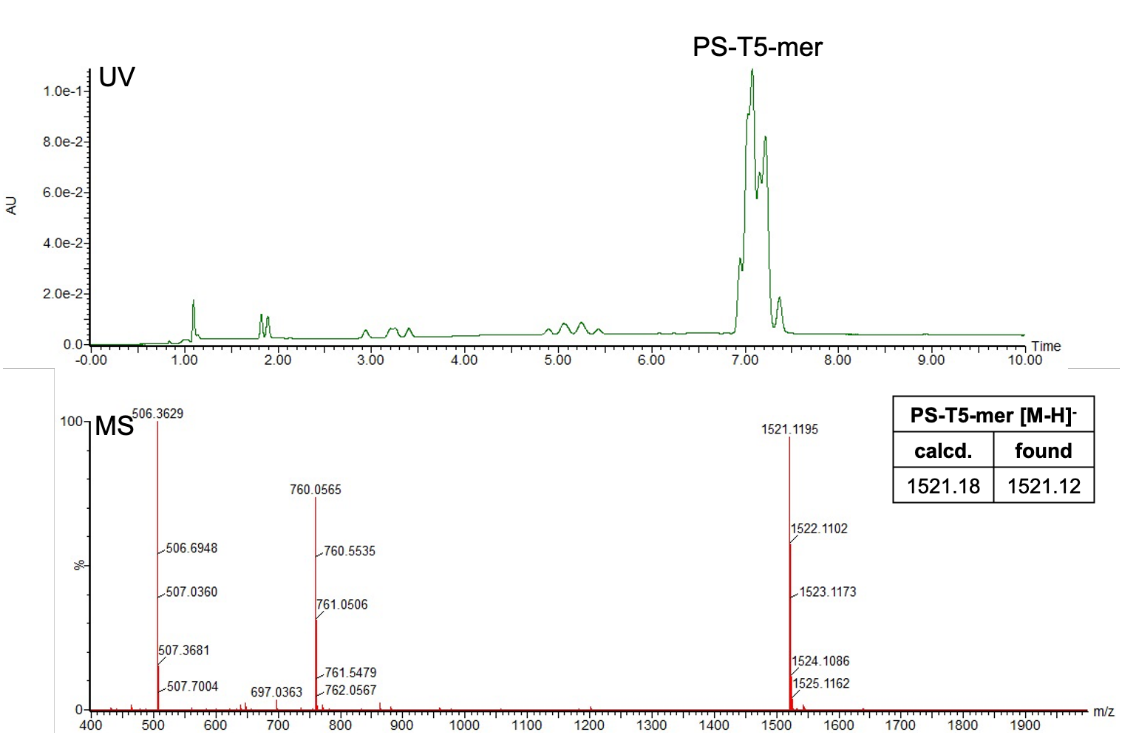

Fig. S33 LC (UV) and MS charts of crude PS-T5-mer synthesised by acetyl capping method (THF without BHT) (Table 3, entry 4).

5) LC (UV) and MS charts of entry 1-4 (Nusinersen) in Table 3

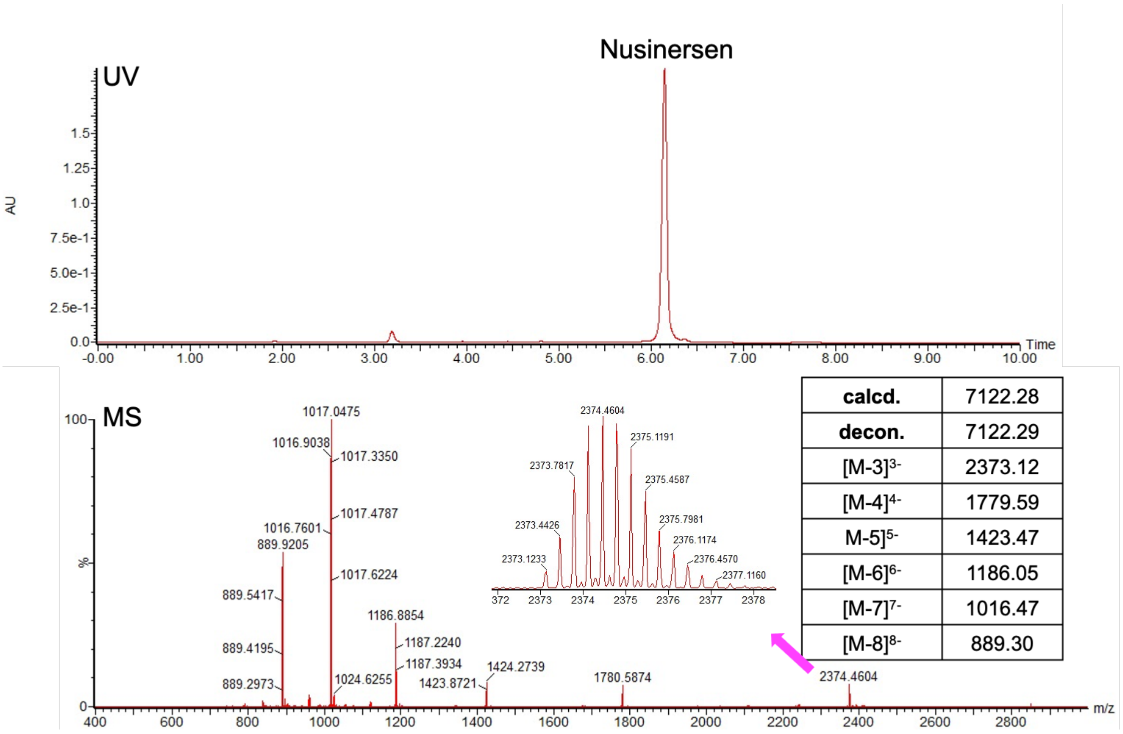

calcd.	7122.28
decon.	7122.29
$[M-3]^{3-}$	2373.12
$[M-4]^{4-}$	1779.59
$M-5]^{5-}$	1423.47
$[M-6]^{6-}$	1186.05
$[M-7]^{7-}$	1016.47
$[M-8]^{8-}$	889.30

Fig. S34 LC (UV) and MS charts of crude nusinersen synthesised by acetyl capping method (THF with BHT) (Table 3, entry 1).

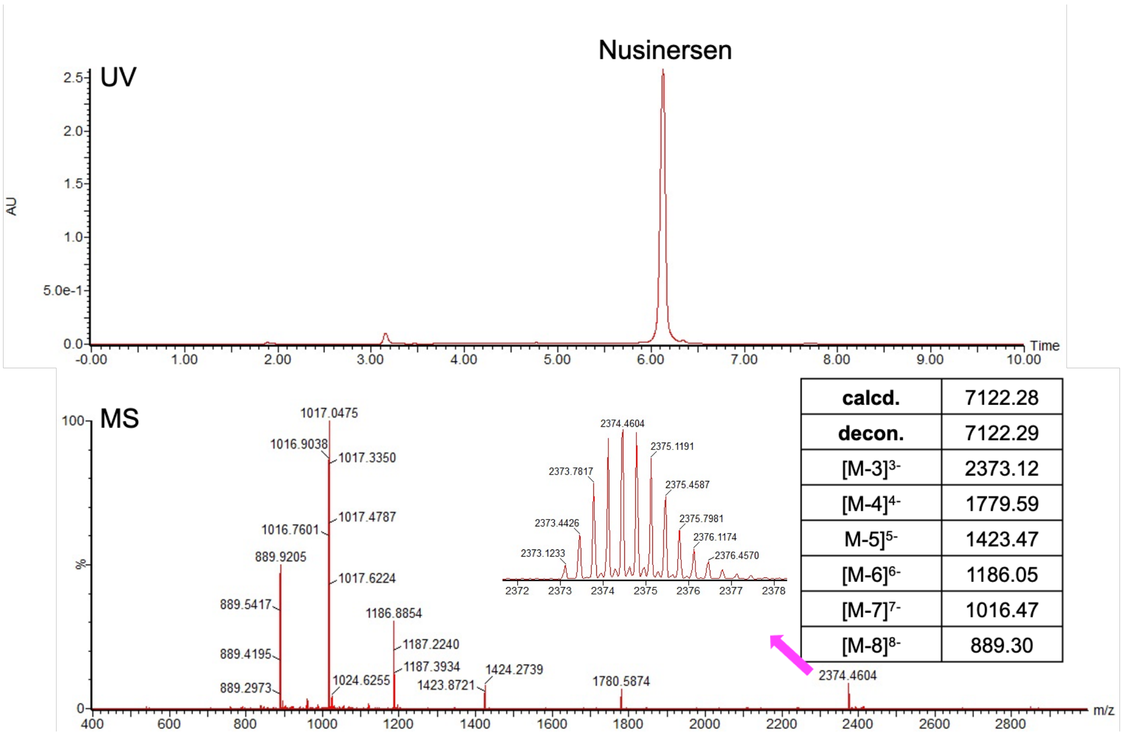

calcd.	7122.28
decon.	7122.29
$[M-3]^{3-}$	2373.12
$[M-4]^{4-}$	1779.59
$M-5]^{5-}$	1423.47
$[M-6]^{6-}$	1186.05
$[M-7]^{7-}$	1016.47
$[M-8]^{8-}$	889.30

Fig. S35 LC (UV) and MS charts of crude nusinersen synthesised by acetyl capping method (THF with BHT) (Table 3, entry 2).

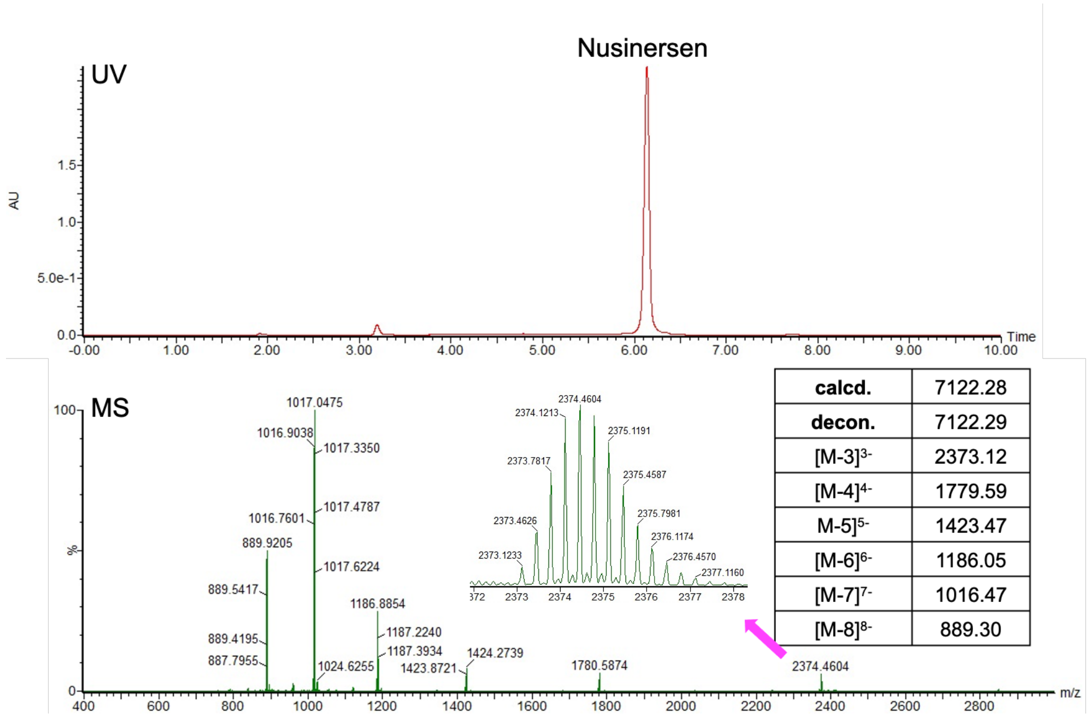

calcd.	7122.28
decon.	7122.29
[M-3]$^{3-}$	2373.12
[M-4]$^{4-}$	1779.59
M-5]$^{5-}$	1423.47
[M-6]$^{6-}$	1186.05
[M-7]$^{7-}$	1016.47
[M-8]$^{8-}$	889.30

Fig. S36 LC (UV) and MS charts of crude nusinersen synthesised by acetyl capping method (THF without BHT) (Table 3, entry 3).

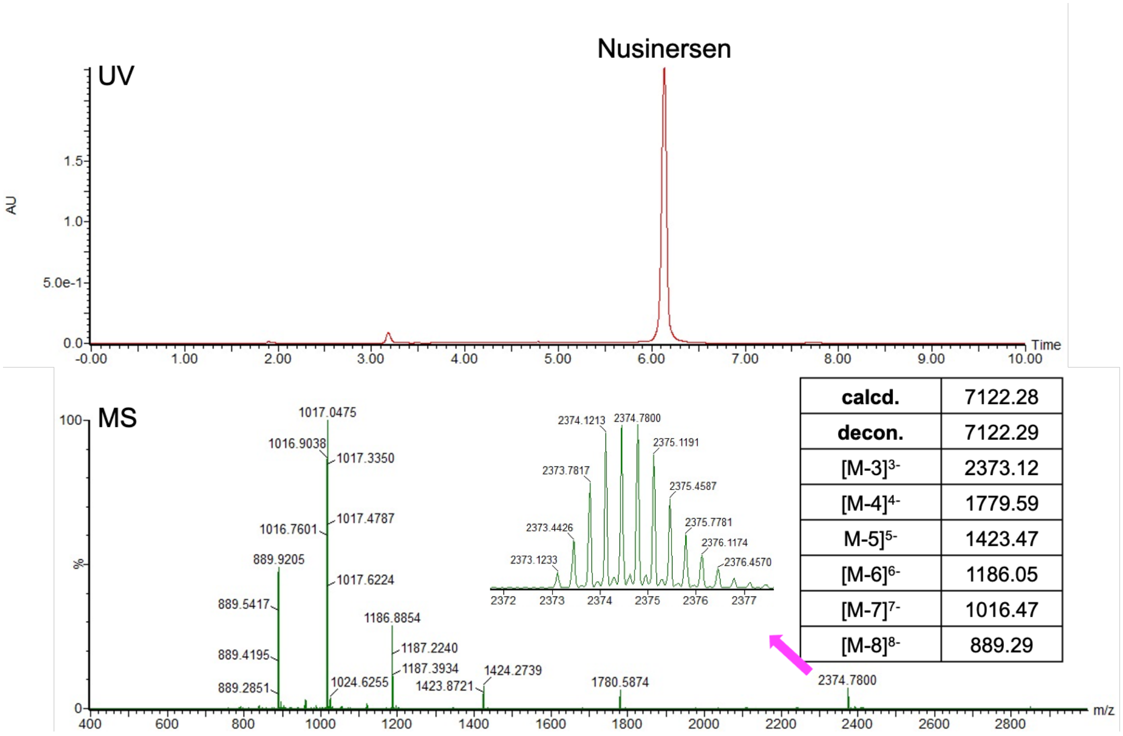

calcd.	7122.28
decon.	7122.29
[M-3]$^{3-}$	2373.12
[M-4]$^{4-}$	1779.59
M-5]$^{5-}$	1423.47
[M-6]$^{6-}$	1186.05
[M-7]$^{7-}$	1016.47
[M-8]$^{8-}$	889.29

Fig. S37 LC (UV) and MS charts of crude nusinersen synthesised by acetyl capping method (THF without BHT) (Table 3, entry 4).

6) LC (UV) and MS charts of entry 1-2 (PS-T5-mer) in Table 4

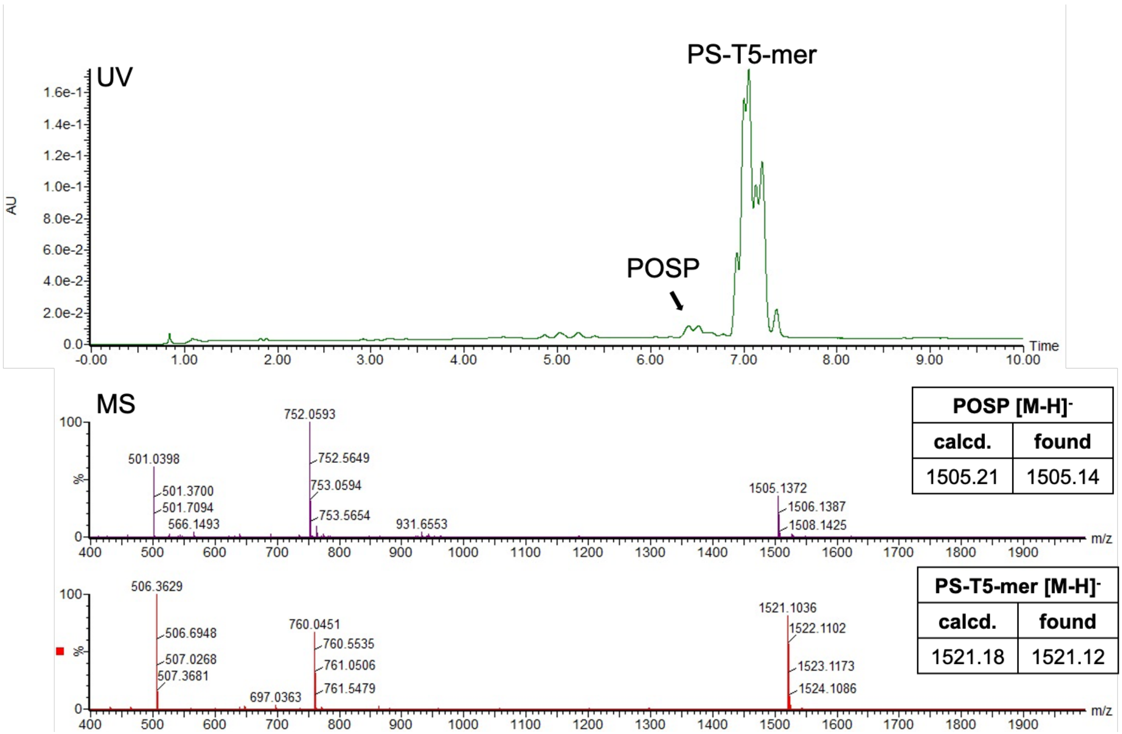

Fig. S38 LC (UV) and MS charts of crude PS-T5-mer synthesised by cholesterol capping method (THF without BHT) (Table 4, entry 1).

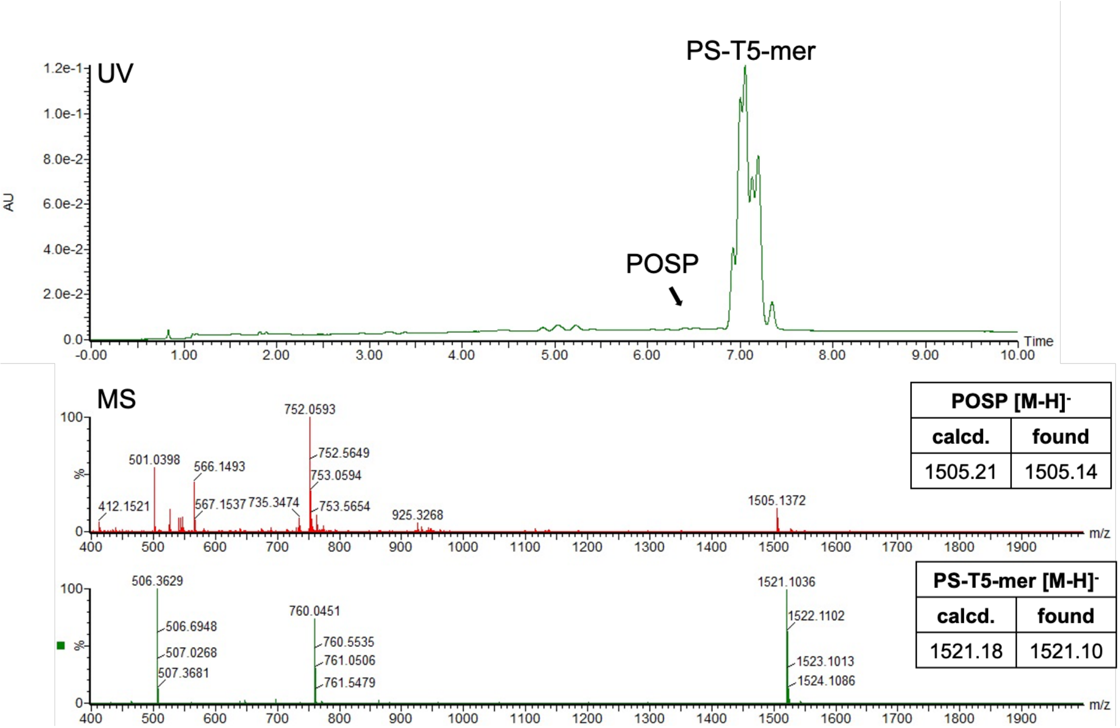

Fig. S39 LC (UV) and MS charts of crude PS-T5-mer synthesised by cholesterol capping method (THF with BHT) (Table 4, entry 2).

7) LC (UV) and MS charts of entry 1-2 (Nusinersen) in Table 4

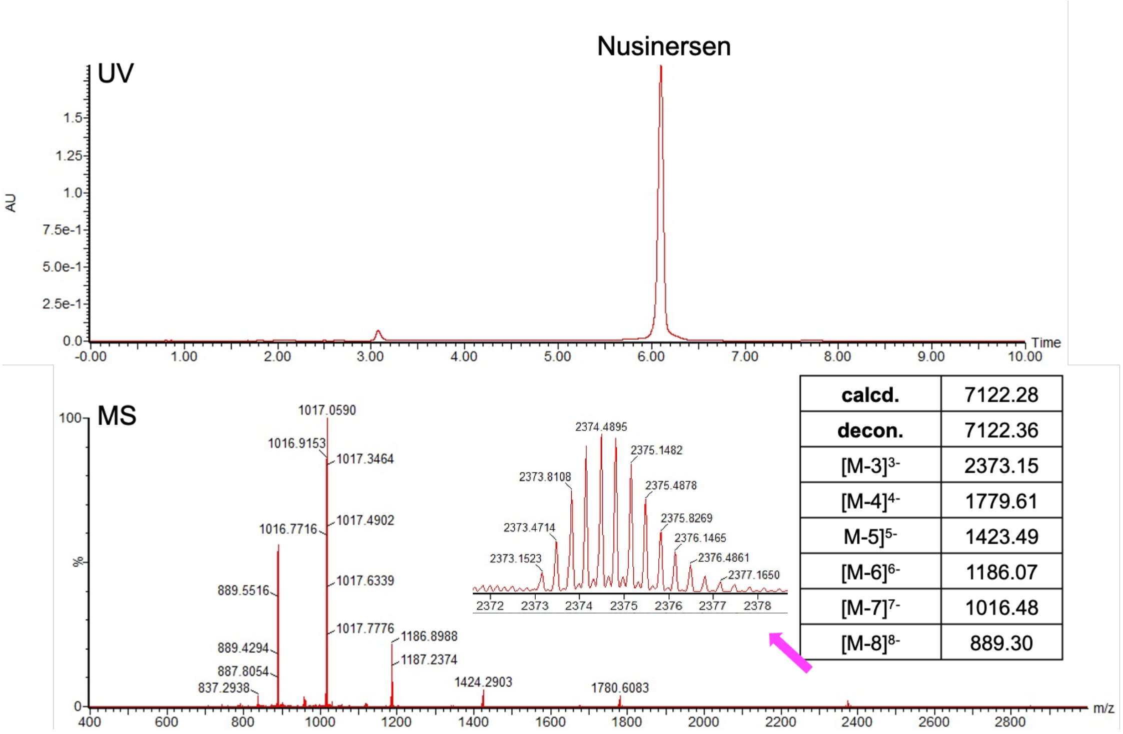

Fig. S40 LC (UV) and MS charts of crude nusinersen synthesised by cholesterol capping method (THF without BHT) (Table 4, entry 1).

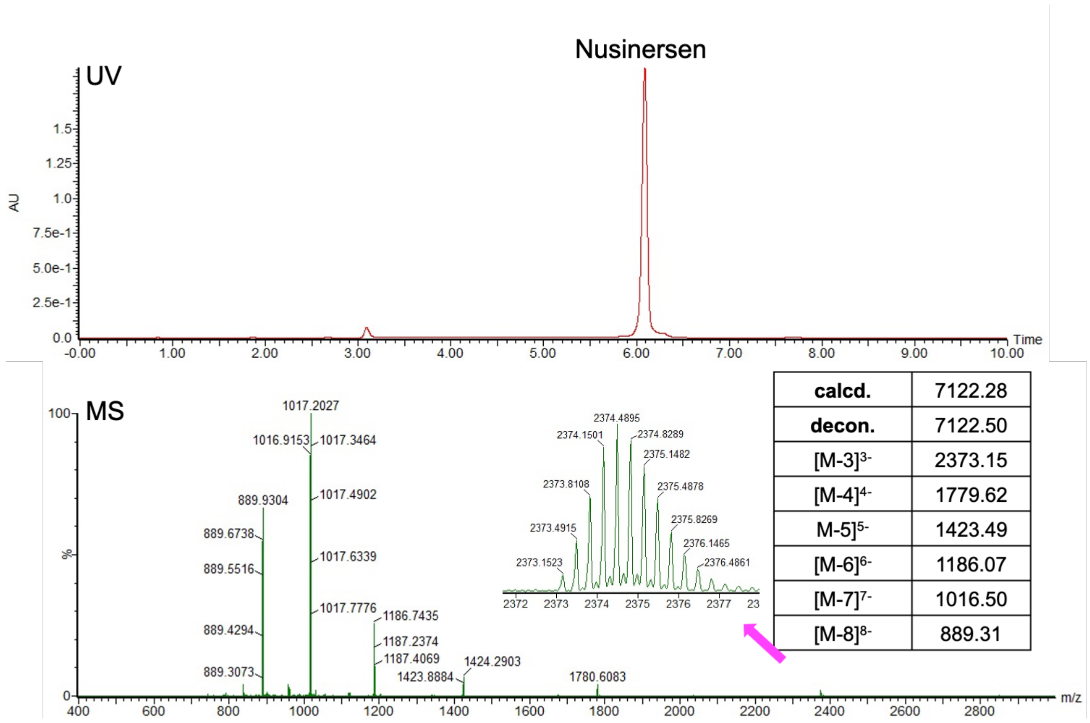

Fig. S41 LC (UV) and MS charts of crude nusinersen synthesised by cholesterol capping method (THF with BHT) (Table 4, entry 2).
